# Supplementary material for: Transforming Palmyra Atoll to native-tree dominance will increase net carbon storage and reduce dissolved organic carbon reef runoff
Source: PLoS One. 2022 Jan 21;17(1):e0262621. doi: 10.1371/journal.pone.0262621 (PMC8782295; doi:10.1371/journal.pone.0262621)
Supplement: S1 Table — Community type was assigned based on the value of the overlapping raster cell. Soil carbon percentages for each sample were binned as high (>20%), medium (10–20%), and low (<10%). Dry bulk density was assigned using the mean values collected in the field. (DOCX) [file pone.0262621.s001.docx]

**S1 Table.** **List of soil samples and data used in random forest model.** Community type was assigned based on the value of the overlapping raster cell. Soil carbon percentages for each sample were binned as high (>20%), medium (10 – 20%), and low (<10%). Dry bulk density was assigned using the mean values collected in the field.

| **Island** | **Community Type** | **Organic Carbon Percentage** | **Sample Year** | **Organic Carbon Percentage Bin** | **Dry Bulk Density** |
| --- | --- | --- | --- | --- | --- |
| Aviation | *C. nucifera* | 6.57 | 2016 | Low | 1 |
| Aviation | *C. nucifera* | 1.92 | 2016 | Low | 1 |
| Aviation | *P. grandis* | 3.10 | 2016 | Low | 1 |
| Cooper | *C. nucifera* | 7.52 | 2019 | Low | 1 |
| Cooper | *C. nucifera* | 1.71 | 2019 | Low | 1 |
| Cooper | *C. nucifera* | 1.29 | 2019 | Low | 1 |
| Cooper | *C. nucifera* | 0.71 | 2019 | Low | 1 |
| Cooper | *C. nucifera* | 0.80 | 2019 | Low | 1 |
| Cooper | *C. nucifera* | 3.32 | 2019 | Low | 1 |
| Cooper | *C. nucifera/H. foertherianum* | 3.40 | 2016 | Low | 1 |
| Cooper | *C. nucifera/H. foertherianum* | 5.83 | 2016 | Low | 1 |
| Cooper | *C. nucifera/H. foertherianum* | 2.73 | 2016 | Low | 1 |
| Cooper | *H. foertherianum-S. sericea* | 5.33 | 2019 | Low | 1 |
| Cooper | *L. repens var. palmyrensis-F cymosa* | 2.76 | 2016 | Low | 1 |
| Cooper | *L. repens var. palmyrensis-F cymosa* | 4.24 | 2016 | Low | 1 |
| Cooper | *P. tectorius* | 3.93 | 2019 | Low | 1 |
| Cooper | *P. tectorius* | 3.98 | 2019 | Low | 1 |
| Cooper | *P. tectorius* | 1.58 | 2019 | Low | 1 |
| Cooper | *P. tectorius* | 3.86 | 2019 | Low | 1 |
| Cooper | *P. tectorius* | 7.99 | 2016 | Low | 1 |
| Cooper | *S. sericea-H. foertherianum* | 2.78 | 2019 | Low | 1 |
| Dudley | *P. grandis/H. foertherianum* | 9.90 | 2019 | Low | 1 |
| Dudley | *P. grandis/H. foertherianum* | 10.70 | 2019 | Med | 0.427 |
| Dudley | *P. grandis/H. foertherianum* | 23.96 | 2019 | High | 0.272 |
| Dudley | *P. grandis/H. foertherianum* | 37.52 | 2019 | High | 0.272 |
| Dudley | *P. grandis/H. foertherianum* | 3.25 | 2016 | Low | 1 |
| Dudley | *P. grandis/H. foertherianum* | 9.93 | 2016 | Low | 1 |
| Dudley | *P. grandis/H. foertherianum* | 13.42 | 2016 | Med | 0.427 |
| Eastern | *P. grandis* | 4.93 | 2019 | Low | 1 |
| Eastern | *P. grandis* | 2.16 | 2019 | Low | 1 |
| Eastern | *P. grandis* | 1.97 | 2019 | Low | 1 |
| Eastern | *P. grandis* | 0.48 | 2016 | Low | 1 |
| Eastern | *P. grandis* | 1.55 | 2016 | Low | 1 |
| Eastern | *P. grandis* | 36.44 | 2016 | High | 0.272 |
| Eastern | *P. grandis* | 9.45 | 2016 | Low | 1 |
| Engineer | *C. nucifera/H. foertherianum* | 4.70 | 2016 | Low | 1 |
| Engineer | *C. nucifera/H. foertherianum* | 7.18 | 2016 | Low | 1 |
| Engineer | *C. nucifera/H. foertherianum* | 30.67 | 2016 | High | 0.272 |
| Engineer | *C. nucifera/H. foertherianum* | 2.13 | 2016 | Low | 1 |
| Engineer | *C. nucifera/H. foertherianum* | 9.07 | 2016 | Low | 1 |
| Engineer | *C. nucifera/H. foertherianum* | 15.95 | 2016 | Med | 0.427 |
| Engineer | *C. nucifera/H. foertherianum* | 1.79 | 2016 | Low | 1 |
| Fern | *C. nucifera* | 3.76 | 2016 | Low | 1 |
| Fern | *C. nucifera* | 38.46 | 2016 | High | 0.272 |
| Holei | *C. nucifera* | 1.26 | 2016 | Low | 1 |
| Holei | *C. nucifera* | 1.96 | 2016 | Low | 1 |
| Holei | *C. nucifera* | 0.91 | 2016 | Low | 1 |
| Holei | *P. grandis* | 2.04 | 2016 | Low | 1 |
| Kaula | *C. nucifera* | 4.90 | 2016 | Low | 1 |
| Kaula | *C. nucifera* | 0.87 | 2016 | Low | 1 |
| Kaula | *P. grandis* | 3.46 | 2016 | Low | 1 |
| Kaula | *P. grandis* | 4.65 | 2016 | Low | 1 |
| Kaula | *P. grandis* | 4.97 | 2016 | Low | 1 |
| Lesley | *P. grandis/H. foertherianum* | 12.58 | 2019 | Med | 0.427 |
| Lesley | *P. grandis/H. foertherianum* | 24.13 | 2019 | High | 0.272 |
| Lesley | *P. grandis/H. foertherianum* | 21.80 | 2016 | High | 0.272 |
| Lesley | *P. grandis/H. foertherianum* | 11.50 | 2016 | Med | 0.427 |
| Lesley | *P. grandis/H. foertherianum* | 8.78 | 2016 | Low | 1 |
| Lesley | *P. grandis/H. foertherianum* | 4.66 | 2016 | Low | 1 |
| N. Fighter | *C. nucifera/H. foertherianum* | 2.82 | 2016 | Low | 1 |
| N. Fighter | *C. nucifera/H. foertherianum* | 4.68 | 2016 | Low | 1 |
| N. Fighter | *C. nucifera/H. foertherianum* | 2.71 | 2016 | Low | 1 |
| N. Fighter | *P. tectorius* | 4.94 | 2016 | Low | 1 |
| N. Fighter | *P. tectorius* | 2.91 | 2016 | Low | 1 |
| Paradise | *C. nucifera* | 1.82 | 2016 | Low | 1 |
| Paradise | *C. nucifera* | 2.50 | 2016 | Low | 1 |
| Paradise | *C. nucifera* | 1.53 | 2016 | Low | 1 |
| Paradise | *C. nucifera/H. foertherianum* | 3.53 | 2016 | Low | 1 |
| Paradise | *P. grandis* | 0.90 | 2016 | Low | 1 |
| Pelican | *H. tiliaceus* | 1.16 | 2016 | Low | 1 |
| Pelican | *H. tiliaceus* | 14.40 | 2016 | Med | 0.427 |
| Pelican | *P. grandis* | 0.79 | 2016 | Low | 1 |
| Portsmouth | *P. grandis/H. foertherianum* | 2.43 | 2016 | Low | 1 |
| S. Fighter | *C. nucifera/H. foertherianum* | 3.06 | 2016 | Low | 1 |
| S. Fighter | *C. nucifera/H. foertherianum* | 2.93 | 2016 | Low | 1 |
| S. Fighter | *H. foertherianum-S. sericea* | 3.93 | 2016 | Low | 1 |
| S. Fighter | *P. tectorius* | 4.38 | 2016 | Low | 1 |
| S. Fighter | *P. tectorius* | 5.57 | 2016 | Low | 1 |
| Sand | *P. grandis* | 4.44 | 2019 | Low | 1 |
| Sand | *P. grandis* | 6.28 | 2019 | Low | 1 |
| Sand | *P. grandis* | 5.76 | 2019 | Low | 1 |
| Sand | *P. grandis* | 2.67 | 2019 | Low | 1 |
| Sand | *P. grandis* | 11.66 | 2019 | Med | 0.427 |
| Sand | *P. grandis* | 5.12 | 2019 | Low | 1 |
| Sand | *P. grandis* | 10.16 | 2019 | Med | 0.427 |
| Sand | *P. grandis* | 3.68 | 2016 | Low | 1 |
| Sand | *P. grandis* | 5.51 | 2016 | Low | 1 |
| Sand | *P. grandis* | 4.94 | 2016 | Low | 1 |
| Sand | *P. grandis* | 4.93 | 2016 | Low | 1 |
| Strawn | *C. nucifera* | 1.47 | 2016 | Low | 1 |
| Strawn | *C. nucifera* | 1.23 | 2016 | Low | 1 |
| Strawn | *P. tectorius* | 3.53 | 2016 | Low | 1 |
| Strawn | *P. tectorius* | 3.05 | 2016 | Low | 1 |
| Strawn | *P. tectorius* | 2.31 | 2016 | Low | 1 |
| Whipporwill | *H. foertherianum-S. sericea* | 15.52 | 2016 | Med | 0.427 |
| Whipporwill | *P. grandis* | 2.17 | 2016 | Low | 1 |
| Whipporwill | *P. grandis* | 1.64 | 2016 | Low | 1 |
| Whipporwill | *P. grandis* | 1.53 | 2016 | Low | 1 |
| Whipporwill | *P. grandis* | 2.76 | 2016 | Low | 1 |
