# Supplementary material for: Transforming Palmyra Atoll to native-tree dominance will increase net carbon storage and reduce dissolved organic carbon reef runoff
Source: PLoS One. 2022 Jan 21;17(1):e0262621. doi: 10.1371/journal.pone.0262621 (PMC8782295; doi:10.1371/journal.pone.0262621)
Supplement: S2 Table — Samples with the same sample ID were sampled from the same individual tree, with each sample (distinguished by sample number) designated individual discs measured as replicates. (DOCX) [file pone.0262621.s002.docx]

**S2 Table**. **Basic wood density samples**. Samples with the same sample ID were sampled from the same individual tree, with each sample (distinguished by sample number) designated individual discs measured as replicates.

| **Sample ID** | **Sample #** | **Species** | **Wood Density (g/cm ^-3^)** |
| --- | --- | --- | --- |
| CI4 | 1 | *H. foertherianum* | 0.35 |
| CI4 | 2 | *H. foertherianum* | 0.27 |
| CI4 | 3 | *H. foertherianum* | 0.30 |
| DI2 | 1 | *H. foertherianum* | 0.28 |
| DI2 | 2 | *H. foertherianum* | 0.32 |
| DI2 | 3 | *H. foertherianum* | 0.27 |
| EI1 | 1 | *H. foertherianum* | 0.26 |
| EI1 | 2 | *H. foertherianum* | 0.37 |
| EI1 | 3 | *H. foertherianum* | 0.44 |
| SI5 | 1 | *H. foertherianum* | 0.25 |
| SI5 | 2 | *H. foertherianum* | 0.33 |
| SI5 | 3 | *H. foertherianum* | 0.31 |
| SI6 | 1 | *H. foertherianum* | 0.23 |
| SI6 | 2 | *H. foertherianum* | 0.23 |
| SI6 | 3 | *H. foertherianum* | 0.26 |
| CI17 | 1 | *P. tectorius* | 0.12 |
| CI17 | 2 | *P. tectorius* | 0.10 |
| CI17 | 3 | *P. tectorius* | 0.12 |
| CI17 | 4 | *P. tectorius* | 0.13 |
| CI17 | 5 | *P. tectorius* | 0.11 |
| CI17 | 6 | *P. tectorius* | 0.13 |
| CI12 | 1 | *P. tectorius* | 0.08 |
| CI12 | 2 | *P. tectorius* | 0.09 |
| CI12 | 3 | *P. tectorius* | 0.10 |
| CI13 | 1 | *P. tectorius* | 0.26 |
| CI13 | 2 | *P. tectorius* | 0.28 |
| CI13 | 3 | *P. tectorius* | 0.30 |
| CI15 | 1 | *P. tectorius* | 0.18 |
| CI15 | 2 | *P. tectorius* | 0.16 |
| CI15 | 3 | *P. tectorius* | 0.17 |
| CI16 | 1 | *P. tectorius* | 0.07 |
| CI16 | 2 | *P. tectorius* | 0.06 |
| CI16 | 3 | *P. tectorius* | 0.09 |
| CI16 | 4 | *P. tectorius* | 0.07 |
| CI16 | 5 | *P. tectorius* | 0.07 |
| CI16 | 6 | *P. tectorius* | 0.10 |
| DI5 | 1 | *P. grandis* | 0.20 |
| DI5 | 2 | *P. grandis* | 0.17 |
| DI5 | 3 | *P. grandis* | 0.20 |
| DI8 | 1 | *P. grandis* | 0.18 |
| DI8 | 2 | *P. grandis* | 0.18 |
| DI8 | 3 | *P. grandis* | 0.17 |
| EI5 | 1 | *P. grandis* | 0.22 |
| EI5 | 2 | *P. grandis* | 0.22 |
| EI5 | 3 | *P. grandis* | 0.21 |
| SI11 | 1 | *P. grandis* | 0.12 |
| SI11 | 2 | *P. grandis* | 0.19 |
| SI11 | 3 | *P. grandis* | 0.22 |
| SI12 | 1 | *P. grandis* | 0.23 |
| SI12 | 2 | *P. grandis* | 0.23 |
| SI12 | 3 | *P. grandis* | 0.21 |
| SI17 | 1 | *P. grandis* | 0.19 |
| SI17 | 2 | *P. grandis* | 0.17 |
| SI17 | 3 | *P. grandis* | 0.17 |
| SI18 | 1 | *P. grandis* | 0.24 |
| SI18 | 2 | *P. grandis* | 0.18 |
| SI18 | 3 | *P. grandis* | 0.18 |
| CI23 | 1 | *S. sericea* | 0.57 |
| CI23 | 2 | *S. sericea* | 0.23 |
| CI23 | 3 | *S. sericea* | 0.38 |
