# Supplementary material for: Transforming Palmyra Atoll to native-tree dominance will increase net carbon storage and reduce dissolved organic carbon reef runoff
Source: PLoS One. 2022 Jan 21;17(1):e0262621. doi: 10.1371/journal.pone.0262621 (PMC8782295; doi:10.1371/journal.pone.0262621)
Supplement: S3 Table — (DOCX) [file pone.0262621.s003.docx]

**S3 Table**. **Summary of quadratic mean of tree diameters for each species used in analysis with the number of trees sampled.**

| **Species** | **Quadratic Mean** | **Number of Samples** |
| --- | --- | --- |
| *C. nucifera* | 28.40 | 104 |
| *C. subcordata* | 11.69 | 7 |
| *H. foertherianum* | 26.07 | 69 |
| *H. tiliaceus* | 21.64 | 17 |
| *P. tectorius* | 17.62 | 63 |
| *P. grandis* | 19.25 | 361 |
| *S. sericea* | 4.76 | 267 |
| *Terminalia catappa* | 26.58 | 63 |
