# Supplementary material for: Transforming Palmyra Atoll to native-tree dominance will increase net carbon storage and reduce dissolved organic carbon reef runoff
Source: PLoS One. 2022 Jan 21;17(1):e0262621. doi: 10.1371/journal.pone.0262621 (PMC8782295; doi:10.1371/journal.pone.0262621)
Supplement: S4 Table — Values used to determine survival rate. (DOCX) [file pone.0262621.s004.docx]

**S4 Table**. **Table depicting results of health surveys for *H. foertherianum* seedling**s. Values used to determine survival rate.

| **Date** | **Plot** | **Seedling** | **Live** | **Dead** |
| --- | --- | --- | --- | --- |
| 10-Nov-19 | 1 | 1 | Y |  |
| 10-Nov-19 | 1 | 2 | Y |  |
| 10-Nov-19 | 1 | 3 |  | Y |
| 10-Nov-19 | 1 | 4 |  | Y |
| 10-Nov-19 | 1 | 5 |  | Y |
| 10-Nov-19 | 1 | 6 |  | Y |
| 10-Nov-19 | 2 | 1 | Y |  |
| 10-Nov-19 | 2 | 2 | Y |  |
| 10-Nov-19 | 2 | 3 | Y |  |
| 10-Nov-19 | 2 | 4 | Y |  |
| 10-Nov-19 | 2 | 5 | Y |  |
| 10-Nov-19 | 2 | 6 | Y |  |
| 10-Nov-19 | 3 | 1 | Y |  |
| 10-Nov-19 | 3 | 2 | Y |  |
| 10-Nov-19 | 3 | 3 | Y |  |
| 10-Nov-19 | 3 | 4 |  | Y |
| 10-Nov-19 | 3 | 5 |  | Y |
| 10-Nov-19 | 3 | 6 |  | Y |
| 10-Nov-19 | 4 | 1 | Y |  |
| 10-Nov-19 | 4 | 2 |  | Y |
| 10-Nov-19 | 4 | 3 |  | Y |
| 10-Nov-19 | 4 | 4 |  | Y |
| 10-Nov-19 | 4 | 5 |  | Y |
| 10-Nov-19 | 4 | 6 |  | Y |
| 10-Nov-19 | 5 | 1 | Y |  |
| 10-Nov-19 | 5 | 2 | Y |  |
| 10-Nov-19 | 5 | 3 | Y |  |
| 10-Nov-19 | 5 | 4 | Y |  |
| 10-Nov-19 | 5 | 5 | Y |  |
| 10-Nov-19 | 5 | 6 | Y |  |
| 10-Nov-19 | 6 | 1 | Y |  |
| 10-Nov-19 | 6 | 2 | Y |  |
| 10-Nov-19 | 6 | 3 | Y |  |
| 10-Nov-19 | 6 | 4 |  | Y |
| 10-Nov-19 | 6 | 5 |  | Y |
| 10-Nov-19 | 6 | 6 |  | Y |
| 10-Nov-19 | 7 | 1 |  | Y |
| 10-Nov-19 | 7 | 2 | Y |  |
| 10-Nov-19 | 7 | 3 | Y |  |
| 10-Nov-19 | 7 | 4 |  | Y |
| 10-Nov-19 | 7 | 5 |  | Y |
| 10-Nov-19 | 7 | 6 |  | Y |
| 10-Nov-19 | 8 | 1 | Y |  |
| 10-Nov-19 | 8 | 2 | Y |  |
| 10-Nov-19 | 8 | 3 | Y |  |
| 10-Nov-19 | 8 | 4 | Y |  |
| 10-Nov-19 | 8 | 5 |  | Y |
| 10-Nov-19 | 8 | 6 |  | Y |
| 10-Nov-19 | 9 | 1 | Y |  |
| 10-Nov-19 | 9 | 2 | Y |  |
| 10-Nov-19 | 9 | 3 | Y |  |
| 10-Nov-19 | 9 | 4 | Y |  |
| 10-Nov-19 | 9 | 5 |  | Y |
| 10-Nov-19 | 9 | 6 |  | Y |
| 10-Nov-19 | 10 | 1 | Y |  |
| 10-Nov-19 | 10 | 2 | Y |  |
| 10-Nov-19 | 10 | 3 | Y |  |
| 10-Nov-19 | 10 | 4 | Y |  |
| 10-Nov-19 | 10 | 5 | Y |  |
| 10-Nov-19 | 10 | 6 |  | Y |
| 10-Nov-19 | 11 | 1 | Y |  |
| 10-Nov-19 | 11 | 2 | Y |  |
| 10-Nov-19 | 11 | 3 | Y |  |
| 10-Nov-19 | 11 | 4 | Y |  |
| 10-Nov-19 | 11 | 5 | Y |  |
| 10-Nov-19 | 11 | 6 |  | Y |
| 10-Nov-19 | 12 | 1 | Y |  |
| 10-Nov-19 | 12 | 2 | Y |  |
| 10-Nov-19 | 12 | 3 | Y |  |
| 10-Nov-19 | 12 | 4 | Y |  |
| 10-Nov-19 | 12 | 5 | Y |  |
| 10-Nov-19 | 12 | 6 |  | Y |
| 10-Nov-19 | 13 | 1 | Y |  |
| 10-Nov-19 | 13 | 2 |  | Y |
| 10-Nov-19 | 13 | 3 | Y |  |
| 10-Nov-19 | 13 | 4 | Y |  |
| 10-Nov-19 | 13 | 5 | Y |  |
| 10-Nov-19 | 13 | 6 |  | Y |
| 10-Nov-19 | 14 | 1 | Y |  |
| 10-Nov-19 | 14 | 2 | Y |  |
| 10-Nov-19 | 14 | 3 | Y |  |
| 10-Nov-19 | 14 | 4 | Y |  |
| 10-Nov-19 | 14 | 5 | Y |  |
| 10-Nov-19 | 14 | 6 |  | Y |
| 10-Nov-19 | 15 | 1 | Y |  |
| 10-Nov-19 | 15 | 2 | Y |  |
| 10-Nov-19 | 15 | 3 | Y |  |
| 10-Nov-19 | 15 | 4 |  | Y |
| 10-Nov-19 | 15 | 5 |  | Y |
| 10-Nov-19 | 15 | 6 |  | Y |
| 10-Nov-19 | 16 | 1 | Y |  |
| 10-Nov-19 | 16 | 2 | Y |  |
| 10-Nov-19 | 16 | 3 |  | Y |
| 10-Nov-19 | 16 | 4 |  | Y |
| 10-Nov-19 | 16 | 5 |  | Y |
| 10-Nov-19 | 16 | 6 |  | Y |
| 10-Nov-19 | 17 | 1 | Y |  |
| 10-Nov-19 | 17 | 2 | Y |  |
| 10-Nov-19 | 17 | 3 | Y |  |
| 10-Nov-19 | 17 | 4 | Y |  |
| 10-Nov-19 | 17 | 5 |  | Y |
| 10-Nov-19 | 17 | 6 |  | Y |
| 10-Nov-19 | 18 | 1 |  | Y |
| 10-Nov-19 | 18 | 2 |  | Y |
| 10-Nov-19 | 18 | 3 |  | Y |
| 10-Nov-19 | 18 | 4 |  | Y |
| 10-Nov-19 | 18 | 5 |  | Y |
| 10-Nov-19 | 18 | 6 |  | Y |
| 10-Nov-19 | 19 | 1 | Y |  |
| 10-Nov-19 | 19 | 2 | Y |  |
| 10-Nov-19 | 19 | 3 | Y |  |
| 10-Nov-19 | 19 | 4 | Y |  |
| 10-Nov-19 | 19 | 5 |  | Y |
| 10-Nov-19 | 19 | 6 |  | Y |
| 10-Nov-19 | 20 | 1 | Y |  |
| 10-Nov-19 | 20 | 2 | Y |  |
| 10-Nov-19 | 20 | 3 | Y |  |
| 10-Nov-19 | 20 | 4 |  | Y |
| 10-Nov-19 | 20 | 5 |  | Y |
| 10-Nov-19 | 20 | 6 |  | Y |
| 10-Nov-19 | 21 | 1 | Y |  |
| 10-Nov-19 | 21 | 2 | Y |  |
| 10-Nov-19 | 21 | 3 | Y |  |
| 10-Nov-19 | 21 | 4 | Y |  |
| 10-Nov-19 | 21 | 5 |  | Y |
| 10-Nov-19 | 21 | 6 |  | Y |
| 10-Nov-19 | 22 | 1 | Y |  |
| 10-Nov-19 | 22 | 2 | Y |  |
| 10-Nov-19 | 22 | 3 | Y |  |
| 10-Nov-19 | 22 | 4 | Y |  |
| 10-Nov-19 | 22 | 5 |  | Y |
| 10-Nov-19 | 22 | 6 |  | Y |
| 2-Dec-19 | 1 | 1 | Y |  |
| 2-Dec-19 | 1 | 2 | Y |  |
| 2-Dec-19 | 1 | 3 |  | Y |
| 2-Dec-19 | 1 | 4 |  | Y |
| 2-Dec-19 | 1 | 5 |  | Y |
| 2-Dec-19 | 1 | 6 |  | Y |
| 2-Dec-19 | 2 | 1 | Y |  |
| 2-Dec-19 | 2 | 2 | Y |  |
| 2-Dec-19 | 2 | 3 | Y |  |
| 2-Dec-19 | 2 | 4 | Y |  |
| 2-Dec-19 | 2 | 5 | Y |  |
| 2-Dec-19 | 2 | 6 |  | Y |
| 2-Dec-19 | 3 | 1 | Y |  |
| 2-Dec-19 | 3 | 2 | Y |  |
| 2-Dec-19 | 3 | 3 | Y |  |
| 2-Dec-19 | 3 | 4 | Y |  |
| 2-Dec-19 | 3 | 5 |  | Y |
| 2-Dec-19 | 3 | 6 |  | Y |
| 2-Dec-19 | 4 | 1 | Y |  |
| 2-Dec-19 | 4 | 2 | Y |  |
| 2-Dec-19 | 4 | 3 |  | Y |
| 2-Dec-19 | 4 | 4 |  | Y |
| 2-Dec-19 | 4 | 5 |  | Y |
| 2-Dec-19 | 4 | 6 |  | Y |
| 2-Dec-19 | 5 | 1 | Y |  |
| 2-Dec-19 | 5 | 2 | Y |  |
| 2-Dec-19 | 5 | 3 | Y |  |
| 2-Dec-19 | 5 | 4 | Y |  |
| 2-Dec-19 | 5 | 5 | Y |  |
| 2-Dec-19 | 5 | 6 | Y |  |
| 2-Dec-19 | 6 | 1 | Y |  |
| 2-Dec-19 | 6 | 2 | Y |  |
| 2-Dec-19 | 6 | 3 | Y |  |
| 2-Dec-19 | 6 | 4 |  | Y |
| 2-Dec-19 | 6 | 5 |  | Y |
| 2-Dec-19 | 6 | 6 |  | Y |
| 2-Dec-19 | 7 | 1 |  | Y |
| 2-Dec-19 | 7 | 2 | Y |  |
| 2-Dec-19 | 7 | 3 | Y |  |
| 2-Dec-19 | 7 | 4 |  | Y |
| 2-Dec-19 | 7 | 5 |  | Y |
| 2-Dec-19 | 7 | 6 |  | Y |
| 2-Dec-19 | 8 | 1 | Y |  |
| 2-Dec-19 | 8 | 2 | Y |  |
| 2-Dec-19 | 8 | 3 | Y |  |
| 2-Dec-19 | 8 | 4 | Y |  |
| 2-Dec-19 | 8 | 5 |  | Y |
| 2-Dec-19 | 8 | 6 |  | Y |
| 2-Dec-19 | 9 | 1 | Y |  |
| 2-Dec-19 | 9 | 2 | Y |  |
| 2-Dec-19 | 9 | 3 | Y |  |
| 2-Dec-19 | 9 | 4 |  | Y |
| 2-Dec-19 | 9 | 5 |  | Y |
| 2-Dec-19 | 9 | 6 |  | Y |
| 2-Dec-19 | 10 | 1 | Y |  |
| 2-Dec-19 | 10 | 2 | Y |  |
| 2-Dec-19 | 10 | 3 | Y |  |
| 2-Dec-19 | 10 | 4 | Y |  |
| 2-Dec-19 | 10 | 5 |  | Y |
| 2-Dec-19 | 10 | 6 |  | Y |
| 2-Dec-19 | 11 | 1 | Y |  |
| 2-Dec-19 | 11 | 2 | Y |  |
| 2-Dec-19 | 11 | 3 | Y |  |
| 2-Dec-19 | 11 | 4 | Y |  |
| 2-Dec-19 | 11 | 5 | Y |  |
| 2-Dec-19 | 11 | 6 |  | Y |
| 2-Dec-19 | 12 | 1 | Y |  |
| 2-Dec-19 | 12 | 2 | Y |  |
| 2-Dec-19 | 12 | 3 | Y |  |
| 2-Dec-19 | 12 | 4 | Y |  |
| 2-Dec-19 | 12 | 5 | Y |  |
| 2-Dec-19 | 12 | 6 |  | Y |
| 2-Dec-19 | 13 | 1 | Y |  |
| 2-Dec-19 | 13 | 2 |  | Y |
| 2-Dec-19 | 13 | 3 | Y |  |
| 2-Dec-19 | 13 | 4 | Y |  |
| 2-Dec-19 | 13 | 5 | Y |  |
| 2-Dec-19 | 13 | 6 |  | Y |
| 2-Dec-19 | 14 | 1 | Y |  |
| 2-Dec-19 | 14 | 2 | Y |  |
| 2-Dec-19 | 14 | 3 | Y |  |
| 2-Dec-19 | 14 | 4 | Y |  |
| 2-Dec-19 | 14 | 5 | Y |  |
| 2-Dec-19 | 14 | 6 |  | Y |
| 2-Dec-19 | 15 | 1 | Y |  |
| 2-Dec-19 | 15 | 2 | Y |  |
| 2-Dec-19 | 15 | 3 | Y |  |
| 2-Dec-19 | 15 | 4 |  | Y |
| 2-Dec-19 | 15 | 5 |  | Y |
| 2-Dec-19 | 15 | 6 |  | Y |
| 2-Dec-19 | 16 | 1 | Y |  |
| 2-Dec-19 | 16 | 2 |  | Y |
| 2-Dec-19 | 16 | 3 |  | Y |
| 2-Dec-19 | 16 | 4 |  | Y |
| 2-Dec-19 | 16 | 5 |  | Y |
| 2-Dec-19 | 16 | 6 |  | Y |
| 2-Dec-19 | 17 | 1 | Y |  |
| 2-Dec-19 | 17 | 2 | Y |  |
| 2-Dec-19 | 17 | 3 |  | Y |
| 2-Dec-19 | 17 | 4 | Y |  |
| 2-Dec-19 | 17 | 5 |  | Y |
| 2-Dec-19 | 17 | 6 |  | Y |
| 2-Dec-19 | 18 | 1 |  | Y |
| 2-Dec-19 | 18 | 2 |  | Y |
| 2-Dec-19 | 18 | 3 |  | Y |
| 2-Dec-19 | 18 | 4 |  | Y |
| 2-Dec-19 | 18 | 5 |  | Y |
| 2-Dec-19 | 18 | 6 |  | Y |
| 2-Dec-19 | 19 | 1 | Y |  |
| 2-Dec-19 | 19 | 2 | Y |  |
| 2-Dec-19 | 19 | 3 | Y |  |
| 2-Dec-19 | 19 | 4 | Y |  |
| 2-Dec-19 | 19 | 5 | Y |  |
| 2-Dec-19 | 19 | 6 |  | Y |
| 2-Dec-19 | 20 | 1 | Y |  |
| 2-Dec-19 | 20 | 2 |  | Y |
| 2-Dec-19 | 20 | 3 |  | Y |
| 2-Dec-19 | 20 | 4 |  | Y |
| 2-Dec-19 | 20 | 5 |  | Y |
| 2-Dec-19 | 20 | 6 |  | Y |
| 2-Dec-19 | 21 | 1 | Y |  |
| 2-Dec-19 | 21 | 2 | Y |  |
| 2-Dec-19 | 21 | 3 |  | Y |
| 2-Dec-19 | 21 | 4 |  | Y |
| 2-Dec-19 | 21 | 5 |  | Y |
| 2-Dec-19 | 21 | 6 |  | Y |
| 2-Dec-19 | 22 | 1 | Y |  |
| 2-Dec-19 | 22 | 2 | Y |  |
| 2-Dec-19 | 22 | 3 | Y |  |
| 2-Dec-19 | 22 | 4 | Y |  |
| 2-Dec-19 | 22 | 5 |  | Y |
| 2-Dec-19 | 22 | 6 |  | Y |
| 30-Dec-19 | 1 | 1 | Y |  |
| 30-Dec-19 | 1 | 2 | Y |  |
| 30-Dec-19 | 1 | 3 |  | Y |
| 30-Dec-19 | 1 | 4 |  | Y |
| 30-Dec-19 | 1 | 5 |  | Y |
| 30-Dec-19 | 1 | 6 |  | Y |
| 30-Dec-19 | 2 | 1 | Y |  |
| 30-Dec-19 | 2 | 2 | Y |  |
| 30-Dec-19 | 2 | 3 | Y |  |
| 30-Dec-19 | 2 | 4 | Y |  |
| 30-Dec-19 | 2 | 5 | Y |  |
| 30-Dec-19 | 2 | 6 |  | Y |
| 30-Dec-19 | 3 | 1 | Y |  |
| 30-Dec-19 | 3 | 2 | Y |  |
| 30-Dec-19 | 3 | 3 | Y |  |
| 30-Dec-19 | 3 | 4 | Y |  |
| 30-Dec-19 | 3 | 5 |  | Y |
| 30-Dec-19 | 3 | 6 |  | Y |
| 30-Dec-19 | 4 | 1 |  | Y |
| 30-Dec-19 | 4 | 2 | Y |  |
| 30-Dec-19 | 4 | 3 | Y |  |
| 30-Dec-19 | 4 | 4 |  | Y |
| 30-Dec-19 | 4 | 5 |  | Y |
| 30-Dec-19 | 4 | 6 |  | Y |
| 30-Dec-19 | 5 | 1 | Y |  |
| 30-Dec-19 | 5 | 2 | Y |  |
| 30-Dec-19 | 5 | 3 | Y |  |
| 30-Dec-19 | 5 | 4 | Y |  |
| 30-Dec-19 | 5 | 5 | Y |  |
| 30-Dec-19 | 5 | 6 | Y |  |
| 30-Dec-19 | 6 | 1 | Y |  |
| 30-Dec-19 | 6 | 2 | Y |  |
| 30-Dec-19 | 6 | 3 | Y |  |
| 30-Dec-19 | 6 | 4 |  | Y |
| 30-Dec-19 | 6 | 5 |  | Y |
| 30-Dec-19 | 6 | 6 |  | Y |
| 30-Dec-19 | 7 | 1 |  | Y |
| 30-Dec-19 | 7 | 2 | Y |  |
| 30-Dec-19 | 7 | 3 | Y |  |
| 30-Dec-19 | 7 | 4 |  | Y |
| 30-Dec-19 | 7 | 5 |  | Y |
| 30-Dec-19 | 7 | 6 |  | Y |
| 30-Dec-19 | 8 | 1 | Y |  |
| 30-Dec-19 | 8 | 2 | Y |  |
| 30-Dec-19 | 8 | 3 | Y |  |
| 30-Dec-19 | 8 | 4 | Y |  |
| 30-Dec-19 | 8 | 5 |  | Y |
| 30-Dec-19 | 8 | 6 |  | Y |
| 30-Dec-19 | 9 | 1 | Y |  |
| 30-Dec-19 | 9 | 2 | Y |  |
| 30-Dec-19 | 9 | 3 | Y |  |
| 30-Dec-19 | 9 | 4 |  | Y |
| 30-Dec-19 | 9 | 5 |  | Y |
| 30-Dec-19 | 9 | 6 |  | Y |
| 30-Dec-19 | 10 | 1 | Y |  |
| 30-Dec-19 | 10 | 2 | Y |  |
| 30-Dec-19 | 10 | 3 | Y |  |
| 30-Dec-19 | 10 | 4 | Y |  |
| 30-Dec-19 | 10 | 5 |  | Y |
| 30-Dec-19 | 10 | 6 |  | Y |
| 30-Dec-19 | 11 | 1 | Y |  |
| 30-Dec-19 | 11 | 2 | Y |  |
| 30-Dec-19 | 11 | 3 | Y |  |
| 30-Dec-19 | 11 | 4 | Y |  |
| 30-Dec-19 | 11 | 5 | Y |  |
| 30-Dec-19 | 11 | 6 | Y |  |
| 30-Dec-19 | 12 | 1 |  | Y |
| 30-Dec-19 | 12 | 2 | Y |  |
| 30-Dec-19 | 12 | 3 | Y |  |
| 30-Dec-19 | 12 | 4 | Y |  |
| 30-Dec-19 | 12 | 5 | Y |  |
| 30-Dec-19 | 12 | 6 |  | Y |
| 30-Dec-19 | 13 | 1 | Y |  |
| 30-Dec-19 | 13 | 2 |  | Y |
| 30-Dec-19 | 13 | 3 | Y |  |
| 30-Dec-19 | 13 | 4 | Y |  |
| 30-Dec-19 | 13 | 5 | Y |  |
| 30-Dec-19 | 13 | 6 |  | Y |
| 30-Dec-19 | 14 | 1 | Y |  |
| 30-Dec-19 | 14 | 2 | Y |  |
| 30-Dec-19 | 14 | 3 | Y |  |
| 30-Dec-19 | 14 | 4 | Y |  |
| 30-Dec-19 | 14 | 5 | Y |  |
| 30-Dec-19 | 14 | 6 |  | Y |
| 30-Dec-19 | 15 | 1 | Y |  |
| 30-Dec-19 | 15 | 2 | Y |  |
| 30-Dec-19 | 15 | 3 | Y |  |
| 30-Dec-19 | 15 | 4 |  | Y |
| 30-Dec-19 | 15 | 5 |  | Y |
| 30-Dec-19 | 15 | 6 |  | Y |
| 30-Dec-19 | 16 | 1 | Y |  |
| 30-Dec-19 | 16 | 2 | Y |  |
| 30-Dec-19 | 16 | 3 |  | Y |
| 30-Dec-19 | 16 | 4 |  | Y |
| 30-Dec-19 | 16 | 5 |  | Y |
| 30-Dec-19 | 16 | 6 |  | Y |
| 30-Dec-19 | 17 | 1 | Y |  |
| 30-Dec-19 | 17 | 2 | Y |  |
| 30-Dec-19 | 17 | 3 |  | Y |
| 30-Dec-19 | 17 | 4 | Y |  |
| 30-Dec-19 | 17 | 5 |  | Y |
| 30-Dec-19 | 17 | 6 |  | Y |
| 30-Dec-19 | 18 | 1 |  | Y |
| 30-Dec-19 | 18 | 2 |  | Y |
| 30-Dec-19 | 18 | 3 |  | Y |
| 30-Dec-19 | 18 | 4 |  | Y |
| 30-Dec-19 | 18 | 5 |  | Y |
| 30-Dec-19 | 18 | 6 |  | Y |
| 30-Dec-19 | 19 | 1 |  | Y |
| 30-Dec-19 | 19 | 2 | Y |  |
| 30-Dec-19 | 19 | 3 | Y |  |
| 30-Dec-19 | 19 | 4 | Y |  |
| 30-Dec-19 | 19 | 5 | Y |  |
| 30-Dec-19 | 19 | 6 |  | Y |
| 30-Dec-19 | 20 | 1 | Y |  |
| 30-Dec-19 | 20 | 2 |  | Y |
| 30-Dec-19 | 20 | 3 |  | Y |
| 30-Dec-19 | 20 | 4 |  | Y |
| 30-Dec-19 | 20 | 5 |  | Y |
| 30-Dec-19 | 20 | 6 |  | Y |
| 30-Dec-19 | 21 | 1 | Y |  |
| 30-Dec-19 | 21 | 2 | Y |  |
| 30-Dec-19 | 21 | 3 |  | Y |
| 30-Dec-19 | 21 | 4 |  | Y |
| 30-Dec-19 | 21 | 5 |  | Y |
| 30-Dec-19 | 21 | 6 |  | Y |
| 30-Dec-19 | 22 | 1 | Y |  |
| 30-Dec-19 | 22 | 2 | Y |  |
| 30-Dec-19 | 22 | 3 | Y |  |
| 30-Dec-19 | 22 | 4 | Y |  |
| 30-Dec-19 | 22 | 5 |  | Y |
| 30-Dec-19 | 22 | 6 |  | Y |
| 3-Feb-20 | 1 | 1 | Y |  |
| 3-Feb-20 | 1 | 2 | Y |  |
| 3-Feb-20 | 1 | 3 |  | Y |
| 3-Feb-20 | 1 | 4 |  | Y |
| 3-Feb-20 | 1 | 5 |  | Y |
| 3-Feb-20 | 1 | 6 |  | Y |
| 3-Feb-20 | 2 | 1 | Y |  |
| 3-Feb-20 | 2 | 2 | Y |  |
| 3-Feb-20 | 2 | 3 | Y |  |
| 3-Feb-20 | 2 | 4 | Y |  |
| 3-Feb-20 | 2 | 5 | Y |  |
| 3-Feb-20 | 2 | 6 |  | Y |
| 3-Feb-20 | 3 | 1 | Y |  |
| 3-Feb-20 | 3 | 2 | Y |  |
| 3-Feb-20 | 3 | 3 | Y |  |
| 3-Feb-20 | 3 | 4 | Y |  |
| 3-Feb-20 | 3 | 5 |  | Y |
| 3-Feb-20 | 3 | 6 |  | Y |
| 3-Feb-20 | 4 | 1 |  | Y |
| 3-Feb-20 | 4 | 2 |  | Y |
| 3-Feb-20 | 4 | 3 | Y |  |
| 3-Feb-20 | 4 | 4 |  | Y |
| 3-Feb-20 | 4 | 5 |  | Y |
| 3-Feb-20 | 4 | 6 |  | Y |
| 3-Feb-20 | 5 | 1 | Y |  |
| 3-Feb-20 | 5 | 2 | Y |  |
| 3-Feb-20 | 5 | 3 | Y |  |
| 3-Feb-20 | 5 | 4 | Y |  |
| 3-Feb-20 | 5 | 5 | Y |  |
| 3-Feb-20 | 5 | 6 | Y |  |
| 3-Feb-20 | 6 | 1 | Y |  |
| 3-Feb-20 | 6 | 2 |  | Y |
| 3-Feb-20 | 6 | 3 |  | Y |
| 3-Feb-20 | 6 | 4 | Y |  |
| 3-Feb-20 | 6 | 5 | Y |  |
| 3-Feb-20 | 6 | 6 |  | Y |
| 3-Feb-20 | 7 | 1 | Y |  |
| 3-Feb-20 | 7 | 2 | Y |  |
| 3-Feb-20 | 7 | 3 | Y |  |
| 3-Feb-20 | 7 | 4 |  | Y |
| 3-Feb-20 | 7 | 5 |  | Y |
| 3-Feb-20 | 7 | 6 |  | Y |
| 3-Feb-20 | 8 | 1 | Y |  |
| 3-Feb-20 | 8 | 2 | Y |  |
| 3-Feb-20 | 8 | 3 | Y |  |
| 3-Feb-20 | 8 | 4 | Y |  |
| 3-Feb-20 | 8 | 5 |  | Y |
| 3-Feb-20 | 8 | 6 |  | Y |
| 3-Feb-20 | 9 | 1 | Y |  |
| 3-Feb-20 | 9 | 2 | Y |  |
| 3-Feb-20 | 9 | 3 | Y |  |
| 3-Feb-20 | 9 | 4 | Y |  |
| 3-Feb-20 | 9 | 5 |  | Y |
| 3-Feb-20 | 9 | 6 |  | Y |
| 3-Feb-20 | 10 | 1 | Y |  |
| 3-Feb-20 | 10 | 2 | Y |  |
| 3-Feb-20 | 10 | 3 | Y |  |
| 3-Feb-20 | 10 | 4 | Y |  |
| 3-Feb-20 | 10 | 5 |  | Y |
| 3-Feb-20 | 10 | 6 |  | Y |
| 3-Feb-20 | 11 | 1 | Y |  |
| 3-Feb-20 | 11 | 2 | Y |  |
| 3-Feb-20 | 11 | 3 | Y |  |
| 3-Feb-20 | 11 | 4 | Y |  |
| 3-Feb-20 | 11 | 5 | Y |  |
| 3-Feb-20 | 11 | 6 | Y |  |
| 3-Feb-20 | 12 | 1 |  | Y |
| 3-Feb-20 | 12 | 2 | Y |  |
| 3-Feb-20 | 12 | 3 | Y |  |
| 3-Feb-20 | 12 | 4 | Y |  |
| 3-Feb-20 | 12 | 5 | Y |  |
| 3-Feb-20 | 12 | 6 |  | Y |
| 3-Feb-20 | 13 | 1 | Y |  |
| 3-Feb-20 | 13 | 2 | Y |  |
| 3-Feb-20 | 13 | 3 | Y |  |
| 3-Feb-20 | 13 | 4 | Y |  |
| 3-Feb-20 | 13 | 5 | Y |  |
| 3-Feb-20 | 13 | 6 |  | Y |
| 3-Feb-20 | 14 | 1 | Y |  |
| 3-Feb-20 | 14 | 2 | Y |  |
| 3-Feb-20 | 14 | 3 | Y |  |
| 3-Feb-20 | 14 | 4 | Y |  |
| 3-Feb-20 | 14 | 5 | Y |  |
| 3-Feb-20 | 14 | 6 |  | Y |
| 3-Feb-20 | 15 | 1 | Y |  |
| 3-Feb-20 | 15 | 2 | Y |  |
| 3-Feb-20 | 15 | 3 | Y |  |
| 3-Feb-20 | 15 | 4 |  | Y |
| 3-Feb-20 | 15 | 5 |  | Y |
| 3-Feb-20 | 15 | 6 |  | Y |
| 3-Feb-20 | 16 | 1 | Y |  |
| 3-Feb-20 | 16 | 2 | Y |  |
| 3-Feb-20 | 16 | 3 |  | Y |
| 3-Feb-20 | 16 | 4 |  | Y |
| 3-Feb-20 | 16 | 5 |  | Y |
| 3-Feb-20 | 16 | 6 |  | Y |
| 3-Feb-20 | 17 | 1 | Y |  |
| 3-Feb-20 | 17 | 2 | Y |  |
| 3-Feb-20 | 17 | 3 |  | Y |
| 3-Feb-20 | 17 | 4 | Y |  |
| 3-Feb-20 | 17 | 5 |  | Y |
| 3-Feb-20 | 17 | 6 |  | Y |
| 3-Feb-20 | 18 | 1 |  | Y |
| 3-Feb-20 | 18 | 2 |  | Y |
| 3-Feb-20 | 18 | 3 |  | Y |
| 3-Feb-20 | 18 | 4 |  | Y |
| 3-Feb-20 | 18 | 5 |  | Y |
| 3-Feb-20 | 18 | 6 |  | Y |
| 3-Feb-20 | 19 | 1 | Y |  |
| 3-Feb-20 | 19 | 2 | Y |  |
| 3-Feb-20 | 19 | 3 | Y |  |
| 3-Feb-20 | 19 | 4 | Y |  |
| 3-Feb-20 | 19 | 5 | Y |  |
| 3-Feb-20 | 19 | 6 |  | Y |
| 3-Feb-20 | 20 | 1 | Y |  |
| 3-Feb-20 | 20 | 2 |  | Y |
| 3-Feb-20 | 20 | 3 |  | Y |
| 3-Feb-20 | 20 | 4 |  | Y |
| 3-Feb-20 | 20 | 5 |  | Y |
| 3-Feb-20 | 20 | 6 |  | Y |
| 3-Feb-20 | 21 | 1 | Y |  |
| 3-Feb-20 | 21 | 2 | Y |  |
| 3-Feb-20 | 21 | 3 |  | Y |
| 3-Feb-20 | 21 | 4 |  | Y |
| 3-Feb-20 | 21 | 5 |  | Y |
| 3-Feb-20 | 21 | 6 |  | Y |
| 3-Feb-20 | 22 | 1 | Y |  |
| 3-Feb-20 | 22 | 2 | Y |  |
| 3-Feb-20 | 22 | 3 | Y |  |
| 3-Feb-20 | 22 | 4 | Y |  |
| 3-Feb-20 | 22 | 5 |  | Y |
| 3-Feb-20 | 22 | 6 |  | Y |
