# Supplementary material for: Transforming Palmyra Atoll to native-tree dominance will increase net carbon storage and reduce dissolved organic carbon reef runoff
Source: PLoS One. 2022 Jan 21;17(1):e0262621. doi: 10.1371/journal.pone.0262621 (PMC8782295; doi:10.1371/journal.pone.0262621)
Supplement: S5 Table — Islet areas are included. (DOCX) [file pone.0262621.s005.docx]

**S5 Table.** **Tables of pre and post transformation carbon values by islet.** Islet areas are included.

| **Islet** | **Area (ha)** | **Current Soil Carbon (Mg C)** | **Current Aboveground Carbon (Mg C)** | **Projected Soil Carbon (Mg C)** | **Projected Aboveground Carbon (Mg C)** |
| --- | --- | --- | --- | --- | --- |
| Ainsley | 0.02 | 0.50 | 0.04 | 0.50 | 0.04 |
| Aviation | 4.28 | 296.81 | 27.35 | 412.76 | 9.48 |
| Barren | 3.83 | 234.20 | 4.20 | 234.20 | 4.20 |
| Bird | 0.26 | 7.53 | 0.85 | 7.53 | 0.85 |
| Bunker | 0.12 | 11.36 | 0.27 | 11.78 | 0.22 |
| Cooper | 91.81 | 5,014.22 | 236.43 | 5,361.23 | 171.28 |
| Dudley | 0.89 | 86.97 | 1.12 | 86.26 | 1.10 |
| Eastern | 11.43 | 695.26 | 17.98 | 703.78 | 16.15 |
| East-West Causeway | 0.23 | 4.52 | 1.84 | 4.52 | 1.71 |
| Engineer | 6.52 | 418.65 | 46.79 | 580.52 | 22.42 |
| Fern | 0.25 | 16.42 | 2.85 | 24.88 | 1.52 |
| Holei | 11.42 | 760.44 | 53.65 | 771.33 | 50.11 |
| Home | 0.71 | 63.85 | 4.95 | 62.45 | 4.95 |
| Kaula | 14.18 | 893.84 | 105.32 | 1,403.70 | 32.82 |
| Lesley | 0.61 | 56.69 | 1.11 | 54.39 | 0.87 |
| Lost | 0.19 | 20.56 | 0.24 | 19.38 | 0.23 |
| Marine | 5.50 | 344.77 | 45.87 | 564.43 | 20.66 |
| Milky Way | 0.09 | 2.34 | 0.68 | 2.34 | 0.56 |
| N. Fighter | 6.13 | 381.12 | 9.68 | 432.40 | 6.29 |
| North-South Causeway | 1.69 | 80.84 | 11.76 | 80.84 | 11.24 |
| Paradise | 6.21 | 352.29 | 34.97 | 462.32 | 15.60 |
| Pelican | 6.07 | 397.30 | 17.63 | 409.74 | 17.34 |
| Portsmouth | 0.26 | 26.21 | 0.24 | 26.32 | 0.21 |
| Quail | 2.43 | 156.11 | 8.23 | 183.70 | 6.43 |
| S. Fighter | 8.12 | 526.31 | 9.52 | 529.28 | 7.24 |
| Sand | 6.92 | 476.72 | 14.86 | 476.72 | 14.70 |
| Strawn | 7.04 | 474.04 | 30.73 | 611.04 | 12.75 |
| Whipporwill | 1.18 | 72.43 | 3.42 | 72.43 | 2.28 |
