# Supplementary material for: Transforming Palmyra Atoll to native-tree dominance will increase net carbon storage and reduce dissolved organic carbon reef runoff
Source: PLoS One. 2022 Jan 21;17(1):e0262621. doi: 10.1371/journal.pone.0262621 (PMC8782295; doi:10.1371/journal.pone.0262621)
Supplement: S6 Table — Note that the total values will not match those in S5 Table as values were only calculated for the major tree species reported in Table 3. (DOCX) [file pone.0262621.s006.docx]

**S6 Table**. **Tables of pre and post transformation carbon values by major tree species.** Note that the total values will not match those in S5 table as values were only calculated for the major tree species reported in Table 3.

| **Tree species** | **Current Carbon (Mg C)** | **Projected Carbon (Mg C)** |
| --- | --- | --- |
| *C. nucifera* | 5,422.68 | 2,518.14 |
| *H. foertherianum* | 1,613.40 | 3,828.32 |
| *H. tiliaceus* | 524.70 | 167.86 |
| *P. tectorius* | 1,417.02 | 1,739.16 |
| *P. grandis* | 932.69 | 4,216.01 |
| *S. sericea* | 1,726.17 | 556.21 |
| *T. cattapa* | 757.96 | 977.62 |
