# Supplementary material for: Transforming Palmyra Atoll to native-tree dominance will increase net carbon storage and reduce dissolved organic carbon reef runoff
Source: PLoS One. 2022 Jan 21;17(1):e0262621. doi: 10.1371/journal.pone.0262621 (PMC8782295; doi:10.1371/journal.pone.0262621)
Supplement: S1 Fig — Box plot used to evaluate the accuracy of corrected BWD estimates. Our corrected BWD estimates for Pisonia are significantly higher than uncorrected estimates (t-test, p = 0.037) and are indistinguishable from Pisonia BWD estimated outside Palmyra Atoll (t-test, p = 0.12). The BWD estimates from the literature (green), uncorrected Palmyra Atoll (purple) and corrected Palmyra Atoll (red) are shown in the boxplot for each genera of Palmyra Atoll trees. (PDF) [file pone.0262621.s007.pdf]

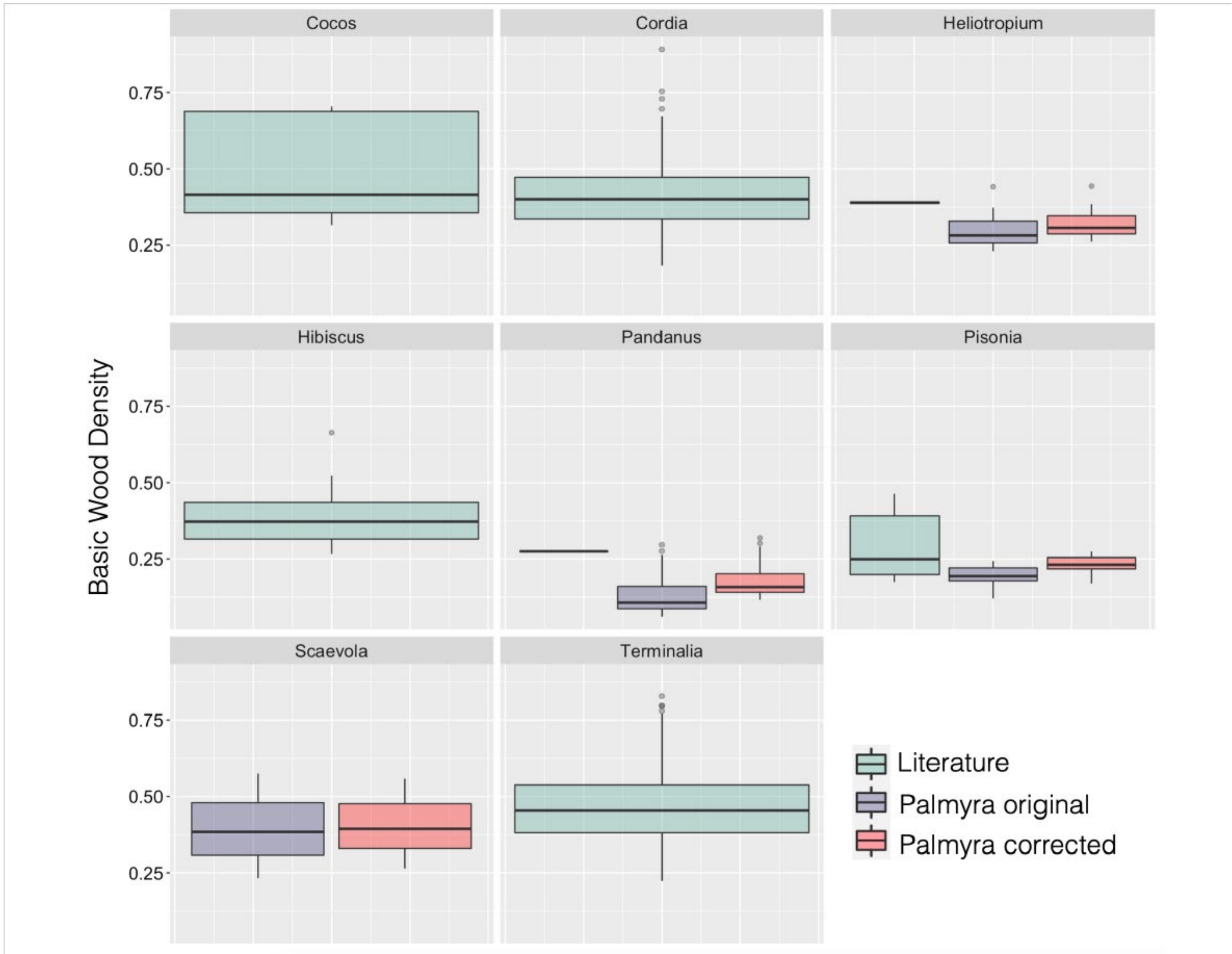

S1 Fig. Basic wood density box plot. Box plot used to evaluate the accuracy of corrected BWD estimates. Our corrected BWD estimates for Pisonia are significantly higher than uncorrected estimates (t-test, $p = 0.037$) and are indistinguishable from Pisonia BWD estimated outside Palmyra Atoll (t-test, $p = 0.12$). The BWD estimates from the literature (green), uncorrected Palmyra Atoll (purple) and corrected Palmyra Atoll (red) are shown in the boxplot for each genera of Palmyra Atoll trees.
