# Supplementary material for: Transforming Palmyra Atoll to native-tree dominance will increase net carbon storage and reduce dissolved organic carbon reef runoff
Source: PLoS One. 2022 Jan 21;17(1):e0262621. doi: 10.1371/journal.pone.0262621 (PMC8782295; doi:10.1371/journal.pone.0262621)
Supplement: Attachment — Submitted filename: PONE-D-21-19703_reviewer-1.pdf [file pone.0262621.s012.pdf]

PLOS ONE

Transforming Palmyra Atoll’s forest to native-tree dominance is predicted to reduce above ground biomass and carbon export to the ocean, yet increase net carbon storage by elevating soil carbon

--Manuscript Draft--

**Manuscript Number:**	PONE-D-21-19703
**Article Type:**	Research Article
**Full Title:**	Transforming Palmyra Atoll’s forest to native-tree dominance is predicted to reduce above ground biomass and carbon export to the ocean, yet increase net carbon storage by elevating soil carbon
**Short Title:**	Impacts of forest transformation activities on Palmyra Atoll’s carbon cycle
**Corresponding Author:**	Kate Longley-Wood, M.Sc.<br>The Nature Conservancy<br>Boston, Massachusetts UNITED STATES
**Keywords:**	Conservation, Pisonia, coconut palm, carbon, dissolved organic carbon, Palmyra
**Abstract:**	Native forests on tropical islands have been displaced by non-native species, leading to calls for their transformation. Simultaneously, there is increasing recognition that tropical forests can help sequester carbon that would otherwise enter the atmosphere. However, it is not always clear whether native forests sequester more or less carbon than human-altered landscapes. At Palmyra Atoll, efforts are underway to transform the rainforest composition from coconut palm (Cocos nucifera) dominated to native mixed-species. To help predict how this landscape-level change might affect the atoll’s carbon cycle, we used field sampling, remote sensing, and parameter estimates from the literature to model the total carbon sequestration potential of Palmyra’s forest before and after transformation. Field measurements included the first known basic wood density measurements for Pisonia grandis , a native tree species declining throughout the tropical Pacific. The model predicted that replacing the C. nucifera plantation with native species would reduce aboveground biomass from 505.5 to 336.5 Mg C. However, the expansion of the native P. grandis and Heliotropium foertherianum forest community was predicted to increase soil carbon to at least 11,856.19 Mg C, increasing the atoll’s overall terrestrial carbon storage potential by at least 12.5%. Nearshore sites adjacent to C. nucifera canopy had a higher dissolved organic carbon (DOC) concentration (110.0 μMC) than sites adjacent to native forest (81.5 μMC), suggesting that, in conjunction with an increase in terrestrial carbon storage, replacing C. nucifera with native forest will reduce the DOC exported from the forest into in nearshore marine habitats. Reduced DOC has potentially positive benefits for corals and coral dependent communities. For tropical islands like Palmyra, reverting from C. nucifera dominance to native tree dominance could buffer projected climate change impacts by increasing carbon storage and reducing coral disease.
**Order of Authors:**	Kate Longley-Wood, M.Sc.
	Mary Engels
	Kevin D Lafferty
	Alex Wegmann
**Additional Information:**	
**Question**	**Response**
**Financial Disclosure**<br><br>Enter a financial disclosure statement that describes the sources of funding for the work included in this submission. Review the submission guidelines for detailed requirements. View published research	This project was made possible with funding from the Wildlife Conservation Society Climate Adaptation Fund (https://www.wcsclimateadaptationfund.org/)—established by a grant from the Doris Duke Charitable Foundation and a grant from the National Science Foundation. Funding received by AW in 2018 (no grant number provided). The funders had no role in study design, data collection and analysis, decision to publish, or preparation of the manuscript.

Powered by Editorial Manager® and ProduXion Manager® from Aries Systems Corporation

articles from *PLOS ONE* for specific examples.<br><br>This statement is required for submission and **will appear in the published article** if the submission is accepted. Please make sure it is accurate.<br><br>**Unfunded studies**<br>Enter: *The author(s) received no specific funding for this work.*<br><br>**Funded studies**<br>Enter a statement with the following details:<br>• Initials of the authors who received each award<br>• Grant numbers awarded to each author<br>• The full name of each funder<br>• URL of each funder website<br>• Did the sponsors or funders play any role in the study design, data collection and analysis, decision to publish, or preparation of the manuscript?<br>• **NO** - Include this sentence at the end of your statement: *The funders had no role in study design, data collection and analysis, decision to publish, or preparation of the manuscript.*<br>• **YES** - Specify the role(s) played.<br><br>* typeset	
**Competing Interests**<br><br>Use the instructions below to enter a competing interest statement for this submission. On behalf of all authors, disclose any competing interests that could be perceived to bias this work—acknowledging all financial support and any other relevant financial or non-financial competing interests.<br><br>This statement is required for submission and **will appear in the published article** if the submission is accepted. Please make sure it is accurate and that any funding sources listed in your Funding Information later in the submission form are also declared in your Financial Disclosure statement.	The authors have declared that no competing interests exist.

View published research articles from [*PLOS ONE*] for specific examples.<br><br>**NO authors have competing interests**<br><br>Enter: *The authors have declared that no competing interests exist.*<br><br>**Authors with competing interests**<br><br>Enter competing interest details beginning with this statement:<br><br>*I have read the journal's policy and the authors of this manuscript have the following competing interests: [insert competing interests here]*<br><br>* typeset	
**Ethics Statement**<br><br>Enter an ethics statement for this submission. This statement is required if the study involved:<br><br>• Human participants<br>• Human specimens or tissue<br>• Vertebrate animals or cephalopods<br>• Vertebrate embryos or tissues<br>• Field research<br><br>Write "N/A" if the submission does not require an ethics statement.<br><br>General guidance is provided below. Consult the submission guidelines for detailed instructions. **Make sure that all information entered here is included in the Methods section of the manuscript.**	Research reported here was conducted under USFWS Special Use Permits 12533-19013 and 12533-16007. No protected species were sampled.

**Format for specific study types**<br><br>**Human Subject Research (involving human participants and/or tissue)**<br>• Give the name of the institutional review board or ethics committee that approved the study<br>• Include the approval number and/or a statement indicating approval of this research<br>• Indicate the form of consent obtained (written/oral) or the reason that consent was not obtained (e.g. the data were analyzed anonymously)<br><br>**Animal Research (involving vertebrate animals, embryos or tissues)**<br>• Provide the name of the Institutional Animal Care and Use Committee (IACUC) or other relevant ethics board that reviewed the study protocol, and indicate whether they approved this research or granted a formal waiver of ethical approval<br>• Include an approval number if one was obtained<br>• If the study involved *non-human primates*, add *additional details* about animal welfare and steps taken to ameliorate suffering<br>• If anesthesia, euthanasia, or any kind of animal sacrifice is part of the study, include briefly which substances and/or methods were applied<br><br>**Field Research**<br><br>Include the following details if this study involves the collection of plant, animal, or other materials from a natural setting:<br>• Field permit number<br>• Name of the institution or relevant body that granted permission	
**Data Availability**<br><br>Authors are required to make all data underlying the findings described fully available, without restriction, and from the time of publication. PLOS allows rare exceptions to address legal and ethical concerns. See the PLOS Data Policy and FAQ for detailed information.	Yes - all data are fully available without restriction

A Data Availability Statement describing where the data can be found is required at submission. Your answers to this question constitute the Data Availability Statement and **will be published in the article**, if accepted.<br><br>**Important:** Stating 'data available on request from the author' is not sufficient. If your data are only available upon request, select 'No' for the first question and explain your exceptional situation in the text box.<br><br>Do the authors confirm that all data underlying the findings described in their manuscript are fully available without restriction?	
**Describe where the data may be found in full sentences. If you are copying our sample text, replace any instances of XXX with the appropriate details.**<br><br>• If the data are **held or will be held in a public repository**, include URLs, accession numbers or DOIs. If this information will only be available after acceptance, indicate this by ticking the box below. For example: *All XXX files are available from the XXX database (accession number(s) XXX, XXX.)*.<br>• If the data are all contained **within the manuscript and/or Supporting Information files**, enter the following: *All relevant data are within the manuscript and its Supporting Information files.*<br>• If neither of these applies but you are able to provide **details of access elsewhere**, with or without limitations, please do so. For example:<br><br>*Data cannot be shared publicly because of [XXX]. Data are available from the XXX Institutional Data Access / Ethics Committee (contact via XXX) for researchers who meet the criteria for access to confidential data.*<br><br>*The data underlying the results presented in the study are available from (include the name of the third party*	All relevant data are within the manuscript and its Supporting Information files.

and contact information or URL).<br>• This text is appropriate if the data are owned by a third party and authors do not have permission to share the data.<br><br>* typeset	
Additional data availability information:	

Powered by Editorial Manager® and ProduXion Manager® from Aries Systems Corporation

Manuscript

Click here to access/download;Manuscript;Palmyra_Carbon_Manuscript_Sub

TITLE

Transforming Palmyra Atoll's forest to native-tree dominance is predicted to reduce above ground biomass and carbon export to the ocean, yet increase net carbon storage by elevating soil carbon

SHORT TITLE

Impacts of forest transformation activities on Palmyra Atoll's carbon ~~cycle~~

AUTHORS

Kate Longley-Wood[1*], Mary Engels[2], Kevin D. Lafferty[3], Alex Wegmann[4],

[1]The Nature Conservancy, Protect Oceans Land and Water Program, 99 Bedford St, Suite 500, Boston, MA 02111, USA, kate.longley-wood@tnc.org

[2], Department of Natural Resources and Society, University of Idaho, 875 Perimeter Drive MS 1139, Moscow, ID 83844-1139

[3] U.S. Geological Survey, Western Ecological Research Center, at Marine Science Institute, UC Santa Barbara, CA 93106

[4]The Nature Conservancy, Hawaii, 923 Nu'uanu Ave., Honolulu, HI 96817, USA

*Corresponding author

E-mail: Kate.Longley-Wood@tnc.org

AUTHOR CONTRIBUTIONS

KLW: Data curation, formal analysis, investigation, methodology, project administration, visualization, writing – original draft preparation

ME: Data curation, formal analysis, investigation, methodology, validation, writing – review and editing

KDL: Data curation, formal analysis, investigation, methodology, writing – review and editing

AW: Conceptualization, funding acquisition, project administration, resources, supervision, writing, review and editing

Abstract

Native forests on tropical islands have been displaced by non-native species, leading to calls for their transformation. Simultaneously, there is increasing recognition that tropical forests can help sequester carbon that would otherwise enter the atmosphere. However, it is not always clear whether native forests sequester more or less carbon than human-altered landscapes. At Palmyra Atoll, efforts are underway to transform the rainforest composition from coconut palm (*Cocos nucifera*) dominated to native mixed-species. To help predict how this landscape-level change might affect the atoll's carbon cycle, we used field sampling, remote sensing, and parameter estimates from the literature to model the total carbon sequestration potential of Palmyra's forest before and after transformation. Field measurements included the first known basic wood density measurements for *Pisonia grandis*, a native tree species declining throughout the tropical Pacific. The model predicted that replacing the *C. nucifera* plantation with native species would reduce aboveground biomass from 505.5 to 336.5 Mg C. However, the expansion of the native *P. grandis* and *Heliotropium foertherianum* forest community was predicted to increase soil carbon to at least 11,856.19 Mg C, increasing the atoll's overall terrestrial carbon storage potential by at least 12.5%. Nearshore sites adjacent to *C. nucifera* canopy had a higher dissolved organic carbon (DOC) concentration (110.0 µMC) than sites adjacent to native forest (81.5 µMC), suggesting that, in conjunction with an increase in terrestrial carbon storage, replacing *C. nucifera* with native forest will reduce the DOC exported from the forest into in nearshore marine habitats. Reduced DOC has potentially positive benefits for corals and coral dependent communities. For tropical islands like Palmyra, reverting from *C. nucifera* dominance to native tree dominance could buffer projected climate change impacts by increasing carbon storage and reducing coral disease.

KEY WORD

Conservation, Pisonia, coconut palm, carbon, dissolved organic carbon

Introduction

Planting trees can be a win-win for nature when the land to be restored is unforested, but proposals to replace non-native trees like coconut palms (*Cocos nucifera*) with native forest habitat can lead to societal conflicts and could impact carbon storage and cycling. At Palmyra Atoll ("Palmyra"), efforts are underway to transform the rainforest community composition from *C. nucifera* dominated to native mixed-species. Although Palmyra rainforest management and transformation efforts should have obvious conservation benefits with no societal conflicts, it is less clear how changes in forest composition will alter Palmyra's carbon stock and cycle. This study aims to [illegible]ess whether goals for restoring habitat align with goals for mitigating climate change by modelling the carbon budgets of native and non-native forests and projecting carbon-related impacts of management interventions.

Although relatively isolated from human disturbance, Palmyra, like other island ecosystems [1,2], is impacted by invasive plants [3]. *C. nucifera* has historically been recorded as native vegetation [4]; however, it is unclear whether it has non-native origins [5,6]. Regardless of origin, historical cultivation for copra at Palmyra beginning in the 19th century [4,7,8] and subsequent natural range expansion led to *C. nucifera* occupying ~40% of the forest canopy today [9].

As *C. nucifera* increased, Palmyra's formerly large stands of native forest shrank. The ten tree species native to Palmyra compete with *C. nucifera* for light [10] and freshwater [8,11] and losses to native forests have had indirect impacts on ecosystem dynamics of Palmyra's rainforest. Less food for invertebrate herbivores [12] under the coconut canopy has reduced prey for native geckos [3], the only resident terrestrial vertebrate. Nitrogen and phosphorus deposits are orders of magnitude lower under *C. nucifera* than under the native broadleaf canopy preferred by roosting seabirds [3,13]. In marine environments, the proximity of nutrient-poor palm-dominated forests has been linked to a decrease in plankton abundance and growth, impacting the foraging behavior of manta rays [3,14]. The loss of

native forest cover, especially decline of the iconic atoll native *Pisonia grandis* from competition and invasive scale insect stress [12], coupled with the expansion of *C. nucifera*, motivated efforts to transform Palmyra's rainforest from a palm-dominated canopy to a mixed-species rainforest with native trees and shrubs. Here, we use the term transform, rather than restore because the management objective is not to recreate a predetermined baseline vegetation community, but rather to optimize community conditions to enhance ecological outcomes. Spearheaded by the United States Fish and Wildlife Service (USFWS) and The Nature Conservancy (TNC), these management efforts are expected to take five years and include removal of *C. nucifera* from 95% of the emergent land area and replanting native forest species. ~~*C. nucifera* removal work began in 2019.~~

Forests fix carbon through photosynthesis, and forest biomass (i.e., roots, trunks, branches, and leaves) is approximately 50% carbon [15]. Some of this carbon-rich biomass eventually falls to the forest floor as litter, which then decomposes and adds to soil organic carbon (SOC) [16] .The amount of carbon stored in forest biomass and soils is largely influenced by climatic conditions [15]; however, variations at the species level can also have implications for carbon budgets at the local scale [17,[illegible] Some fraction of SOC is soluble in water and becomes dissolved organic carbon (DOC) [19], which can leach out of the soil into adjacent water bodies. The DOC flux out of a temperate forest ranges between 6% and 30% (average 17%) of aboveground litter input [20]. This rate varies by vegetation type [21], and increases with precipitation, pH, and decomposition [20], environmental conditions that are common at Palmyra. Dissolved organic carbon can affect various processes on coral reefs, including favoring bacterial populations associated with coral disease [22].

Thus, to better understand the carbon implications of the ongoing forest management efforts at Palmyra this study measured existing carbon budgets in Palmyra's current *C. nucifera*-dominated forest and remnant native mixed-species forest. We used field sampling, remote sensing, and parameter estimates from the literature to evaluate above-ground carbon storage, SOC sequestration and DOC

export. This information was then modeled to project how the atoll's carbon budget and export would change under *C. nucifera* removal and replacement with native trees and shrubs. The results from this study may be applicable to other conservation efforts on *C. nucifera*-dominated islands throughout Oceania.

Methods

Estimating soil carbon

Field sampling

Field sampling was conducted within the Palmyra Atoll National Wildlife Refuge (5°52' N, 162°04' W), jointly managed by TNC and USFWS. Research reported here was conducted under USFWS Special Use Permits 12533-19013 and 12533-16007. No protected species were sampled.

To estimate soil carbon storage across Palmyra's dominant tree species, we structured our sampling to account for how islet size and vegetation community, (and by extension, nutrient subsidies) could influence soils. The 20 islets included in this study form a representative subset in terms of relative size: 8 large (>6.5 ha), 5 medium (between 1 and 6.5 ha) and 7 small (<1 ha). In 2016, 72 sampling sites spanning seven canopy types were chosen at random after stratifying by islet and canopy type. Sampling efforts focused on the most widespread and dominant canopy types: *C. nucifera* (N=15), *Pandanus tectorius* (N=15), *Scaevola sericea/Heliotropium foertherianum* (N=15) and *P. grandis* (N=14). Canopy types with more restricted distributions were sampled less: *Terminalia catappa* (N=1), no canopy cover (N=9), and *Hibiscus tiliaceus* (N=3). The replicates for each canopy type came from different islets, except *H. tiliaceus* which had multiple sites from one islet due to its limited distribution.

We augmented the 2016 sampling data in 2019 by adding 27 sites (totaling 99 sites), evening the distribution between samples taken in homogeneous tree communities (n = 47) and mixed-species

communities (n = 52). This was beneficial as mixed tree stands can have different carbon sequestration characteristics than pure stands [23]. This also increased the number of samples collected from islets on the western edge of the lagoon, where dredging activities during the mid-20th century resulted in man-made islets (Sand, Lesley, and Dudley). These islets may have different soil properties and thus different carbon storage capacities than soils occurring on natural islets. Samples from both time periods were assigned a vegetation community type based on remote sensing data (9). 2019 sampling efforts covered the five most dominant woody-stemmed species (*C. nucifera, H. foertherianum, P. tectorius, P. grandis,* and *S. sericea*) on five key islets (Cooper, Eastern, Dudley, Sand, and Lesley Islets). Sampling locations are shown in Figure 1, and Table 1 lists the number of samples by vegetation community type, along with the proportional representation of each community type in the study area. Addit[illegible]l sample data can be found in S1 Table.

Figure 1. Study area with restoration plots and sampling locations and years for soil organic carbon (SOC), basic wood density (BWD) and dissolved organic carbon (DOC).

Table 1. Soil samples by vegetation community, and proportional representation of each community type across Palmyra's vegetated area.

Vegetation community	% Vegetated Area	# of Samples
C. nucifera	29.8%	20
C. nucifera/H. foertherianum	11.5%	16
H. foerthenarium/S. sericea	4.0%	3
H. tiliaceus	2.6%	2
Lepturus repens var. palmyrensis/Fimbristylis cymosa	6.5%	2
P. tectorius	13.0%	12
P.grandis	13.2%	29
P.grandis/H. foertherianum	1.4%	14
S. sericea/H. foertherianum	11.8%	1
Other	6.3%	0

At each sampling location, 1,000 – 2,000 ml of soil were removed from the first 0-15 cm of soil with a small trowel and stored in a sealable plastic bag. Because the coarse textured nature of atoll soils makes traditional coring difficult, we followed the water method for sample volume (24), which determines volume by lining the sample hole with a thin plastic layer and recording the volume of water needed to fill the hole to the reference surface. Hardpan depth at each site was measured with a soil probe. After collection, soil samples were passed through a 2 mm sieve, air-dried, and then the <2 mm fraction was shipped to a laboratory for carbon analysis.

The samples collected in 2016 were analyzed for both organic carbon and total carbon percentage. Although the 2019 samples were analyzed for total carbon only, the organic carbon fraction for the 2019 samples were estimated based on the relationship between total carbon and organic carbon derived from the 2016 samples and samples collected in other atoll environments.

Modelling approach

To estimate soil carbon values across the study area we used the 'randomForest' statistical package in R [25,26] to predict soil carbon percentages using 1 m resolution remotely-sensed vegetation community and tree species dataset as predictor variables. These variables were selected due to the high-resolution spatial data coverage across the study area. The "Woody Crowns Palmyra Atoll 2016" dataset [9] from which the tree species raster was derived contained overlapping polygons, and each pixel in the resulting raster was assigned the tree species value that was most prevalent in the 1 m cell. Similarly, the vegetation community raster was derived by converting the "Vegetation Communities Palmyra Atoll 2016" polygons [9] to a 1 m raster. Before running the model, soil carbon percentages for each sample were binned as high (>20%), medium (10 – 20%), and low (<10%), informed by the Natural Resource Conservation Service organic/mineral soil definitions [27]. Total carbon was then calculated by multiplying the soil percentage value associated with each category by the mean dry bulk density for

each category (S1 Table), the soil depth (assumed to be 20 cm based on average hardpan depth measurements across all soil sampling locations), and total area.

To verify the results of the random forest model, we also calculated present-day soil carbon using the mean carbon concentration and dry bulk density values based on the combination of vegetation community and species at each location. To do so, we converted the 1m community and tree species rasters to polygons and used a geometric union to create a polygon layer identifying community type and tree species across the study area. We then assigned mean carbon concentrations and dry bulk density values to each polygon by calculating averages for each combination of variables (vegetation community and species) in in the sampling data. Where a combined mean could not be calculated from the data (i.e., no values for a specific species was measured or when $n < 2$ for a combination of community and species), the community mean value was used as a default. Similarly, if there was no value for the community, the species value was used. If neither value was available, the mean of all samples was used. We assigned a value of zero to all areas where either community or species value was blank, as this usually indicated a shoreline polygon overlapping with water, or where either value was either unknown, water, or the runway area on Cooper Islet. We then calculated present day carbon values using the same formula described above.

The presence of man-made islets as part of the atoll system provides a unique opportunity to estimate soil carbon accumulation rates for Palmyra's vegetation communities. Dudley and Leslie Islets' primary vegetation community is *P. grandis*/*H. foertherianum*, and the construction of these islets was completed by 1942. Similarly, the North Fighter Strip Islet, home to communities of *H. foertherianum/C. nucifera* was likely completed by 1943 [28]. We assumed a linear accumulation rate, and estimated the yearly soil accumulation rate for the *P. grandis*/*H. foertherianum* and *H. foertherianum/C. nucifera* communities by dividing the current estimated soil carbon totals on these islets and dividing them by 77 years and 76 years, respectively, using 2019 as the baseline year to indicate the minimum accumulation

rate. As there are no homogeneous *C. nucifera* communities on the man-made islets sampled in this study, a sequestration rate was estimated from available literature [29]. We then compared the projected carbon accumulation over 15 years in a no-transformation scenario to our current transformation scenario, to determine the additional carbon added under the transformation scenario.

Estimating aboveground carbon

Field sampling

At each sampling location, a single woody branch approximately 10 cm in length was removed from each tree and cut into discs. Discs were submerged in water for 24 hours after which the diameter and width of each disc was measured in order to record the water-saturated volume. The samples were then fully dried in a drying oven, and then weighed to calculate the oven dry mass. The oven dry mass divided by the water-saturated volume represents the BWD [30] and the mean BWD for each species was recorded. Due to lack of data availability on other key metrics used to calculate biomass, biomass values were only calculated for the dominant tree species at Palmyra. The list of species used in this calculation, and the BWD values and their sources can be found in Table 2 and measurements for each sample can be found in S2 Table.

Table 2. Basic wood density values by species.

Species	Wood Density (g cm^{-3})	SE	Sample Size (n)	Source
C. nucifera	0.33	--	--	Cirad
C. subcordata	0.35	--	--	Cirad
H. foertherianum	0.29	0.01	15	Field data
H. tiliaceus	0.43	--	--	Cirad
P. tectorius	0.13	0.02	21	Field data
P. grandis	0.19	0.01	21	Field data
S. sericea	0.39	0.1	3	Field data
T. catappa	0.39	--	--	Cirad

The source notes whether the value used in aboveground biomass calculations was found in the literature via the Cirad database (31) or derived through field measurements as part of this study. For values calculated from field measurements, the mean, standard error and sample size is provided.

Modelling approach

We estimated aboveground biomass (AGB) using the equation derived specifically for tropical tree species [32], that incorporates basic wood density (BWD) (ρ), tree height (H), and diameter (D) where $AGB = .0509\ X\ \rho D2H$.

BWD represents the ratio of dry mass over green volume. Mean BWD for many commercially-important tree species can be found in the Cirad wood density database [31]. which was used to assign BWD for *C. nucifera*, *Cordia subcordata, H. tiliaceus, and T. catappa*, using the lowest density value if a range was reported. However, because this metric is not available for many tree species native to Palmyra either in the database or in other literature, wood samples were collected from the branches of *P. grandis*, *H. foertherianum, P. tectorius,* and *S. sericea* in order to measure BWD.

The location, species, and height of each tree was derived from remote sensing data [9,33]. To approximate the point location of each tree trunk, we used the remote sensing dataset representing woody crowns to derive the centroid of each crown polygon. In order to estimate the approximate height of each tree, we then used the elevation data to assign a height to each point, subtracting 2m from each location to account for height above sea level. A gap in the LIDAR data for Engineer Islet resulted in some of the points being assigned a mean height value specific to the species across the study area. Diameters were assigned according to tree species by taking the quadratic mean of measured tree diameters collected during transect surveys (S3 Table). Aboveground biomass values were then calculated using the above-referenced equation and then converted to carbon values using a multiplier of 0.47 [34]. The carbon densit[illegible]g C ha^{-1}) is based on an estimate of areas covered by a canopy (i.e. area that excludes runway, bare ground, grassland, and unknown land cover).

Projecting future soil and aboveground carbon

To estimate the expected carbon storage following transformation activities (allowing for transformed forests to reach maturity), we created spatial data layers for expected future vegetation community values and future tree species values, based on a transformation area polygon dataset provided by Island Conservation [35]. Within the transformation polygons, the objective is to remove all *C. nucifera,* and plant *H. foertherianum* and *P. grandis* at a 2:1 ratio. However, since the survival rate of replanted *H. foertherianum* is estimated at 46% (Table S4), mature, transformed forest communities are expected to have approximately half of the existing *C. nucifera* replaced by *H. foertherianum* and half replaced by *P. grandis* [35]. These forest communities will be considered mature 15 years after they have been planted.

Based on the management plan, we simulated the future community scenario data by selecting areas within the remote sensing data [9] that had a community value of *C. nucifera* and were within the transformation polygons and reclassified them as the mixed *P. grandis*/*H. foertherianum* community. Similarly, all tree species points classified as *C. nucifera* within the transformation areas were randomly reassigned to be either *P. grandis*, or *H. foertherianum*, based on the target 50:50 representation. Heights for newly planted *P. grandis* and *H. foertherianum* were assigned based on mean values for those species across the study area. Using the steps described in previous sections, we then recalculated the aboveground and soil carbon values based on predicted future community and species values.

Dissolved organic carbon

DOC was sampled at 12 sites (Figure 1) in order to measure the difference in DOC export between native and non-native canopies, though this sample size was deemed too small to try to model DOC concentrations under the future transformation scenario. Sites were chosen to represent different parts of the atoll, shorelines facing different directions (N, S, E, W), different canopy types and different *C.*

nucifera removal schedules. Two sample[illegible]ere taken 50 m apart at each site. Three comparison samples (expected to be low in terrestrial influence) were taken from surface waters offshore of the atoll, on the forereef and from lagoon away from shore. Water samples were collected in new quart Ziploc bags with powder free gloves and put on ice after collection. To process DOC, Luer lock syringes were filled with 5% HCl for > 1 hour, then rinsed 2 more times with acid, then rinsed with distilled water before use. Filter holders were similarly soaked in 5% HCl and rinsed. The DOC filtering procedure involved drawing 60 ml of water from the sample bag into a syringe as a rinse and then then drawing a second 60 ml for the sample. A combusted GF75 filter was loaded into a clean 25 mm plastic filter holder and connected to the syringe. The syringe was used to filter 30 ml of sample to rinse an acid-washed collection vial three times. The last 30ml was inserted into the vial and the vial was capped. One filter was used per site. Filtered samples were acidified to pH 2- by adding 50ul 4N HCl, then agitated to mix. Samples were stored and shipped to Dr. Craig Carlson's lab at the University of California, Santa Barbara for analysis. Samples were analyzed via high temperature combustion method on a modified Shimadzu TOC-V or Shimadzu TOC-L using the standardization and referencing approaches described in [36]; detection limit was roughly 1 μmol C L^{-1}.

Results

Soil carbon

The random forest analyses predicted soil carbon storage values comparable to the values calculated using averages from vegetation communities and species measurements. The latter approach predicted baseline values that were comparable (11,856.2 Mg C in the averages method compared to 11,872.2 Mg C in the random forest method) and future values that were somewhat higher than those predicted by the random forest analysis (15,378.2 Mg C in the averages method compared to 13,590.8 in the random

forest method). For this reason, only the results of the random forest model are presented, as they reflect a more conservative estimate for changes in carbon storage.

The results of the soil carbon analyses indicate a 14.5% gain in soil carbon in the post-transformation scenario, representing an increase from 73.4 Mg C ha^{-1} to 84.0 Mg C ha^{-1} when averaged across the study area (Table 3). The model projects a reduction in soil carbon contributions from *C. nucifera*, *H. tiliaceus*, and *S. sericea,* and an increase in soil carbon contributions from *H. foertherianum*, *P. tectorius*, *P. grandis*, and *T. cattapa* in under the transformation scenario (Figure 2a). Kaula Islet (14.2 ha) shows the largest gain, by total tonnage, with an increase of 509.9 Mg C, and the second largest gain by percentage (57.0%) - Cooper Islet (91.8 ha) shows the second largest gain by soil carbon, with an increase of 347.0 Mg C (6.9%). Marine Islet (5.5 ha) has the largest gain by percentage, with a percent increase of 63.7% (217.7 Mg C) (S5 Table). A map of the distribution of soil carbon in the pre and post transformation scenarios and areas of loss and gain can be found in Figures 3a – 3c.

Figure 2. Current and future projections of soil organic carbon, (2a), aboveground carbon (2b), and total carbon (soil and aboveground) (2c) by tree species.

Figure 3. Maps of carbon distribution across the study area in the pre (column 1) and post (column 2) restoration scenarios. The third column shows the net gain and loss of carbon between the two scenarios. Figures 3a – 3c show the results for soil carbon. Figures 3d – 3f show the results for aboveground carbon. Figures 3g – 3i show the sum of the two carbon sources. Units are in Mg C ha^{-1} where the area includes only those areas covered by a canopy (i.e. area that excludes runway, bare ground, grassland, and unknown land cover).

Table 3. Summary of current and projected total carbon, by evaluation method.

Carbon source	Baseline Total (Mg C)	Baseline Density (Mg C ha^{-1})	Future Total (Mg C)	Future Density (Mg C ha^{-1})	% Change to Total
Aboveground	505.5	3.1	336.5	2.1	-33.4%

Soil	11,872.3	73.4	13,590.8	84.0	+14.5%
Total	12,377.8	76.5	13,927.3	86.1	+12.5%

The carbon density (Mg C ha^{-1}) is based on an estimate of areas covered by a canopy (i.e. area that excludes runway, bare ground, grassland, and unknown land cover).

Estimates of current ~~carbon soil~~ content in the *P. grandis*/*H. foertherianum and C. nucifera/H. foertherianum* communities on manmade Dudley, Lesley, and North Fighter Strip Islets suggest a soil carbon accumulation rate of 1.3 Mg Carbon ha^{-1} $year^{-1}$ for *P. grandis*/*H. foertherianum and* 1.0 Mg C ha^{-1} yr^{-1} for *C. nucifera/H. foertherianum.* This suggests that after 15 years, 848.2 additional Mg C will have been added to Palmyra's soil compared to a no-transformation scenario.

Basic wood[illegible]nsity

Mean basic wood density values for *H. foertherianum*, *P. tectorius*, *P. grandis*, and *S. sericea* are reported in Table 2. Values calculated from field data ranged from 0.13 (*P. tectorius*) to 0.39 (*S. sericea*). These values are, overall, lower than the BWD values recorded in the literature for other dominant species at Palmyra.

Aboveground carbon

The results of the analyses indicate a decline of 33.3% in total aboveground carbon in the post-transformation scenario, representing a decrease from 3.1 to 2.1 Mg C ha^{-1} when averaged across the study site (Table 3). On average, *C. nucifera* at Palmyra exceed both *P. grandis* and *H. foertherianum* in height, density, and basic wood density, meaning that their removal and replacement results in a loss of aboveground biomass, and, by extension, aboveground carbon stocks. In the post-transformation scenario, the gain in aboveground carbon storage from the addition of *P. grandis* and *H. foertherianum* do not offset the simultaneous losses in carbon storage from the removal of *C. nucifera* (Figure 2b).

Kaula Islet shows the largest loss, both by percentage and total tonnage, with a decrease of 47 Mg C (63%). Cooper Islet shows the second largest loss by total carbon, with a decrease of 42 Mg C. Aviation

Islet (4.3 ha) has the second largest loss by percentage, with a percent decrease of 59% (Table S5). This is similar to the spatial patterns observed for soil organic carbon increases. A map of the distribution of aboveground carbon in the pre and post transformation scenarios and areas of loss and gain can be found in Figures 3d – 3f.

Overall carbon

Overall, the gains in soil carbon in the post-transformation scenario offset the losses to aboveground carbon, resulting in an overall increase (12.5%) in carbon storage in the post-transformation, representing an increase from 76.5 Mg C ha^{-1} to 86.1 Mg C ha^{-1} when averaged across the study area (Table 3). The large gains in soil carbon storage driven by the increased number of *P. grandis* and *H. foertherianum* eclipse the loss of aboveground carbon driven by the removal of *C. nucifera* (Figure 2c). A map of the distribution of total carbon in the pre and post transformation scenarios and areas of loss and gain can be found in Figures 3g – 3i.

Dissolved Organic Carbon

Intertidal (81.5 µMC) sites that were adjacent to native forest did not differ in DOC from offshore (71.9 µMC), forereef (78.9 µMC), or open water lagoon (73.8 µMC) sites. However, DOC concentrations were significantly higher at intertidal sites adjacent to *C. nucifera* forest than all other habitats sampled. Water depth was not a significant factor after accounting for habitat type. If sites were adjacent to *C. nucifera* there was no difference ($p = 0.9$) in their DOC concentration whether they came from the intertidal lagoon flats (117.0 µMC) or intertidal reef flats (112.7 µMC). We pooled all the intertidal samples adjacent to *C. nucifera* canopy before comparing canopy effects with site as random effect to account for repeated measures at a site. We also excluded Sacia Islet because *C. nucifera* had recently been controlled there. Intertidal sites adjacent to *C. nucifera* canopy had a higher DOC concentration (110.0 µMC) than intertidal sites adjacent to native forest (81.5 µMC, $P = 0.0025$, Figure 4). Note that

Saci1E, where *C. nucifera* had been controlled, had a lower DOC (69.1 µMC), than any site with intact *C. nucifera*, suggesting that the hypothesized *C. nucifera* effect could be rapidly reduced after *C. nucifera* removal (Figure 4).

Figure 4. Map of DOC sampling results. On this map, different shapes symbolize different management actions under which the samples were taken; DOC, (µMC) measured at each site is indicated by a label.

A conceptual illustration of study results and other anticipated transformation and realignment benefits can be found in Figure 5.

Figure 5. Conceptual illustration of study results and other anticipated restoration and realignment benefits (bars not to scale). The panel on the left shows a forest dominated by C. nucifera (current pre-restoration scenario on Palmyra), while the panel on the right shows a forest dominated by native species, following anticipated restoration and realignment. In the pre-restoration scenario, a homogenous, non-native vegetation community consisting of *C. nucifera* is associated with higher aboveground carbon values and lower soil carbon values (bars not to scale). In this scenario, palm fronds and coconut dominate the ground cover, leading to increased dissolved oxygen concentrations negatively impacting the health and diversity of nearby coral reefs. In the post-restoration scenario, a restored, native, mixed-species vegetation community is associated with lower aboveground carbon values, with higher soil carbon values, with a total net increase in carbon storage. In this scenario, seabird and understory diversity also increases, and lower DOC concentrations mean increased coral reef health and biodiversity (Figure credit: Adi Khen).

Discussion

This study is the first known attempt to calculate the carbon stored by vegetation for an entire tropical atoll, and represents a unique opportunity to explore the benefits of transformation beyond biodiversity

and ecosystem health in a manner that can inform management activities in other similar environments. The data collected in this study also provide the first known wood density estimates for several atoll species not currently documented in global databases or other published literature. The remote location and limited research facilities at Palmyra constrained sample sizes and restricted field techniques and more samples taken over more time could yield more robust results. Additionally, measures from many comparable studies incorporate estimates of belowground biomass (e.g., roots), leaf litter, and other sources of biomass such as coconuts. The rapid assessment of carbon based on limited field measurements did not sample belowground biomass, thereby potentially underestimating the amount of carbon found in tree biomass in both forest types. However, this study benefited from high resolution, remotely-sensed imagery, not typically available for such remote locations. These datasets allowed for estimates of aboveground carbon storage to be calculated at the scale of the individual tree, while simultaneously allowing for estimates of soil carbon storage potential based on community level characteristics.

The results of the study confirmed that *C. nucifera* and native forest types differed in their carbon sinks and exports at Palmyra, and that changes in the forest composition at the atoll scale will have an impact on carbon stocks, with decreased carbon in wood, but increased carbon in soil, resulting in a net increase in carbon storage under planned rainforest management activities. Carbon content was in the range of expected values for soils in similar environments but was unique for aboveground carbon. It is challenging to directly compare these soil values to other studies due to the range of data collection techniques, spatial resolutions, depth profiles, and specific methods used to quantify SOC elsewhere. When comparing the soil carbon values to mean soil carbon values from a global soil carbon dataset [37] summarized by ecoregions in the Eastern Indo-Pacific [38], we find that soil carbon content at Palmyra falls along the lower end of the range of values found in similar environments (69.6 – 334.4 Mg C ha^{-1}). However, these global datasets are based on a larger soil depth range (1 meter), so differences based on

soil depth alone would not be surprising, yet also may reflect the unique soil formation processes of atoll environments [39]. There are few comparable studies in similar atoll environments. Organic carbon percentages in Palmyra's soils are in line with expected values of 2 – 20% documented in the literature [39], which looked at soil characteristics across a number of Pacific atolls; however, because these values do not have associated bulk density estimates they cannot be converted to total tonnage as we did at Palmyra.

These new data echo previous research at Palmyra which found higher soil organic carbon in areas of low *C. nucifera* density [40]. *C. nucifera* stands at Palmyra may have higher soil organic carbon than *C. nucifera* monocultures in other locales. For example, in Kerala, India, samples taken at the same depth profile (0-20) showed a carbon stock of 24.81 Mg C ha^{-1}. Although this is much lower than the estimates of soil carbon in *C. nucifera* stands at Palmyra (49.8 Mg C ha^{-1}), this may be due to the fact that there are active *C. nucifera* cultivation practices taking place in Kerala, where *C. nucifera* is strictly a monoculture. At Palmyra, *C. nucifera* can be found in mixed-species stands and plant litter (coconuts and fronds) are not removed. In general, our findings are consistent with previous studies documenting reduction in soil carbon following the conversion of native forests to agricultural plantations, inclusive of *C. nucifera* [e.g., 40,41]. Furthermore, the carbon accumulation rate at Palmyra was between the 1.3 Mg C ha^{-1} yr^{-1} seen during 20 years of tropical forest reforestation [43] and values of up to 5 Mg C ha^{-1} yr^{-1} recorded in other moist tropical environments [44].

At Palmyra *C. nucifera* forests have three times the litter accumulation, and yet only one third the soil organic carbon compared to native forests [8]. The balance between carbon inputs from litter fall and retention measured as soil organic carbon suggests that more carbon is leached from *C. nucifera* forests than from native forests. This might be, in part, due to the higher pH in soils from *C. nucifera* forests [8].

Estimates of aboveground carbon at Palmyra were much lower than the 17 – 255 Mg C Carbon ha^{-1} reported from other tropical forests [45]. In Hawaii, for instance, mean aboveground carbon storage rates were 60 – 130 Mg C ha^{-1}, with, as at Palmyra, non-native plants holding more aboveground carbon than young native plants [46]. In the tropics, aboveground carbon stocks typically exceed SOC [47,48]. The relatively low aboveground biomass at Palmyra may be due to shorter tree heights (e.g., *S. sericea*) and relatively low wood density values in dominant vegetation, especially *P. grandis* and *P. tectorius*, which have softer wood texture than, for example, *H. tiliaceus*.

Although some studies tout the carbon storage benefits of agroforestry projects [49,50], other studies show that variation among individual tree species can impact carbon storage potential in tropical ecosystems [18], and changes from native to non-native vegetation or cultivated environments can reduce carbon stocks [e.g., 50–53]. The results of this study align with the latter studies' results and further emphasize that the carbon storage outcomes of conversion will hinge on the biophysical characteristics of both the individual tree species as well as the vegetation community impacts on soil characteristics.

The increased DOC in nearshore water near *C. nucifera* forests suggests that *C. nucifera* removal could have indirect effects on nearshore communities. Dissolved organic carbon (DOC) released by macroalgae is important in the context of coral reef degradation as it contributes to coral mortality by promoting bacterial metabolism on the coral surface. DOC can be important in oligotrophic systems like Palmyra; its primary effect is to increase bacterial metabolism, which can then be a resource for some invertebrates like sponges [54], but also cause disease in corals [22]. Given the limited sampling effort in this preliminary study, we consider this to be a hypothesis that merits more intensive study before reaching conclusions. We note that the paucity of DOC exported from native forests is opposite to the pattern seen for nitrogen associated with seabird guano from native forests, which might increase both coral growth [55] and phytoplankton productivity, which can support zooplankton populations. Higher

zooplankton abundance adjacent to native forest at Palmyra attracts manta rays, and likely has other food-web effects [14].

Our models rest on the assumption that community characteristics unaltered by transformation activities will remain stable over time. This is especially relevant to our estimates of carbon accumulation rates as these do not account for potential sources of soil carbon degradation. Our calculations of accumulation rates do, to some extent, factor in soil carbon degradation, averaged over time since the initial construction of man-made islands; however, these rates are not likely to remain static over longer time scales and under changing climate regimes. Currently, not enough is known about the drivers of soil carbon degradation at Palmyra to model these predictions more accurately.

C. nucifera presence in, and in many instances dominance of, oceanic island forests, through cultivation (copra) or naturalization (drift dispersal) [6] is widespread. An unreported yet likely significant portion of the world's 439 atolls [56] have experienced loss of native forest habitat through the agricultural introduction of *C. nucifera* [57]. *C. nucifera's* role in human migration and settlement throughout Oceania is notable [58], and control of *C. nucifera* to transform native forest should be balanced with the societal value provided by *C. nucifera* to Pacific Island communities. While the biocultural implications of controlling *C. nucifera* on oceanic islands have not been widely studied [59], the benefits of a landscape dominated by native vegetation are becoming more clear. For atoll ecosystems facing near-term challenges from sea level rise and ocean acidification [60], transformation of forests from *C. nucifera* dominance to native tree dominance could not only enhance ecosystem integrity [3,14,61,62] but also act as a natural climate solution through enhanced carbon banking [63]. This study provides further incentive consider the climate resilience benefits associated with intact atoll ecosystems when planning management activities.

Acknowledgements

This project was made possible with funding from the Wildlife Conservation Society Climate Adaptation Fund—established by a grant from the Doris Duke Charitable Foundation and a grant from the National Science Foundation. On site assistance was provided by Stefan Kropidlowski (USFWS), Nick Holmes (TNC California's Island Resilience Program), and TNC staff operating the TNC research facility at Palmyra. 2016 tree survey and soil sampling design and sampling was conducted by John P McLaughlin, Ana Miller-ter Kuile, Michelle Lee (NSF REU), Taylor Bogar (NSF REU), and Hillary Young (PI) of the University of California, Santa Barbara. Elisa Halewood provided the DOC protocol and analyzed the samples. Adi Khen developed the scientific illustration included in this manuscript. Research reported here was conducted under USFWS Special Use Permits 12533-19013 and 12533-16007. Any use of trade, firm, or product names is for descriptive purposes only and does not imply endorsement by the U.S. Government.

References

1. D'antonio CM, Dudley TL. Biological invasions as agents of change on islands versus mainlands. In: Vitousek PM, Loope, LL, Adsersen H, editors. Islands. Springer, Berlin, Heidelberg; 1995. pp 103 - 121.

2. Vitousek PM. Diversity and biological invasions of oceanic islands. Biodiversity. 1988 Jan 15; 20:181-9.

3. Young HS, Miller-ter Kuile A, McCauley DJ, Dirzo R. Cascading community and ecosystem consequences of introduced coconut palms (Cocos nucifera) in tropical islands. Can J Zool. 2017;95(3):139-48.

4. Dawson EY. Changes in Palmyra Atoll and its vegetation through the activities of man, 1913-1958. Pacific Nat. 1959;1(2).

5. Binggeli P. A taxonomic, biogeographical and ecological overview of invasive woody plants. J Veg Sci. 1996;7:121–4.

6. Arulandoo X, Sritharan K, Subramaniam M. The coconut palm. In: Thomas B, Murray BG, Murphy DJ, editors. Encyclopedia of applied plant sciences, 2nd Edition. Cambridge (MA): Academic Press;

2017. pp. 426–30.

7. Hathaway SA, McEachern AK, Fisher RN. Terrestrial forest management plan for Palmyra Atoll. Reston (VA): US Department of the Interior, US Geological Survey; 2011. Report No.: 2011 - 1007. Prepared for The Nature Conservancy Palmyra Program.

8. Young HS, Raab TK, McCauley DJ, Briggs AA, Dirzo R. The coconut palm, Cocos nucifera, impacts forest composition and soil characteristics at Palmyra Atoll, Central Pacific. J Veg Sci. 2010;21(6):1058–68.

9. Struckhoff M. Woody species crown and vegetation community data, Palmyra Atoll, 2016; 2019 [Cited 2019 Sep 1]. Dataset available from https://www.sciencebase.gov/catalog/item/5c66fc5be4b0fe48cb3aca49

10. Wegmann AS. Limitations to tree seedling recruitment at Palmyra Atoll. Ph.D. Dissertation. University of Hawaii. 2009.

11. Krauss KW, Duberstein JA, Cormier N, Young HS, Hathaway SA. Proximity to encroaching coconut palm limits native forest water use and persistence on a Pacific atoll. Ecohydrology. 2015 Dec 1;8(8):1514–24.

12. Nonner E, Woodward LA. The Decline of Pisonia grandis at Palmyra Atoll NWR; Factors and Solutions. 2006. Report to the US Fish and Wildlife Service.

13. Young HS, McCauley DJ, Dunbar RB, Dirzo R. Plants cause ecosystem nutrient depletion via the interruption of bird-derived spatial subsidies. Proc Natl Acad Sci. 2010 Feb 2;107(5):2072–7.

14. McCauley DJ, Desalles PA, Young HS, Dunbar RB, Dirzo R, Mills MM, et al. From wing to wing: The persistence of long ecological interaction chains in less-disturbed ecosystems. Sci Rep. 2012;2(1): 1-5.

15. Malhi Y, Meir P, Brown S. Forests, carbon and global climate. Philosophical Transactions of the Royal Society A: Mathematical, Physical and Engineering Sciences. Royal Society; 2002 Aug 15; 360(1797): 1567–91.

16. Maraseni TN, Pandey SS. Can vegetation types work as an indicator of soil organic carbon? An insight from native vegetations in Nepal. Ecol Indic. 2014 Nov 1;46:315 - 22.

17. Kirby KR, Potvin C. Variation in carbon storage among tree species: Implications for the management of a small-scale carbon sink project. For Ecol Manage. 2007 Jul 31;246(2–3):208–21.

18. Russell AE, Raich JW, Arrieta RB, Valverde-Barrantes O, González E. Impacts of individual tree species on carbon dynamics in a moist tropical forest environment. Ecol Appl [Internet]. 2010 Jun;20(4):1087–100. Available from: http://doi.wiley.com/10.1890/09-0635.1.

19. Michalzik B, Tipping E, Mulder J, Lancho JFG, Matzner E, Bryant CL, et al. Modelling the production and transport of dissolved organic carbon in forest soils. Biogeochemistry. 2003 Dec;66(3):241-64.

20. Michalzik B, Kalbitz K, Park J-H, Solinger S, Matzner E. Fluxes and concentrations of dissolved organic carbon and nitrogen - a synthesis for temperate forests. Biogeochemistry. 2001;52(2): 173 - 205.

21. Meyer JL, Wallace JB, Eggert SL. Leaf litter as a source of dissolved organic carbon in streams.

Ecosystems [Internet]. 1998 [cited 2020 Nov 2];1(3):240–9. Available from: https://link.springer.com/article/10.1007/s100219900019

22. Kline D, Kuntz N, Breitbart M, Knowlton N, Rohwer F. Role of elevated organic carbon levels and microbial activity in coral mortality. Mar Ecol Prog Ser. 2006 May 22;314: 119 - 25.

23. Redondo-Brenes A, Montagnini F. Growth, productivity, aboveground biomass, and carbon sequestration of pure and mixed native tree plantations in the Caribbean lowlands of Costa Rica. For Ecol Manage. 2006 Aug 15;232(1–3):168–78.

24. Page-Dumroese DS, Brown RE, Jurgensen MF, Mroz GD. Comparison of methods for determining bulk densities of rocky forest soils. Soil Sci Soc Am J. 2010;63(2):379.

25. Liaw A, Wiener M. Classification and regression by randomForest. R News. 2002;2(3):18–22.

26. R: A language and environment for statistical computing. Version 4.0.3 [software]. Vienna, Austria: R Foundation for Statistical Computing. 2020 [cited 15 Jan 2020]. Available from: https://www.r-project.org/

27. Soil Survey Staff. Soil survey field and laboratory methods manual. 51st ed. Burt R, Soil Survey Staff, editors. Lincoln (NE): Department of Agriculture, Natural Resources Conservation Science; 2014.

28. Johnson J. A history lost and found: Research highlights from the Palmyra Atoll digital archive. 2021 Jan. [Cited 2021 May 1]. Available from: www.PalmyraArchive.org.

29. Selvaraj A, Jayachandran S, Thirunavukkarasu DP, Jayaraman A, Karuppan P. Carbon sequestration potential, physicochemical and microbiological properties of selected trees Mangifera indica L., Manilkara zapota L., Cocos nucifera L. and Tectona grandis L. Biosci Discov [Internet]. 2016;7(2):131–9. Available from: http://biosciencediscovery.com

30. Magnussen S, Reed D. Modelling for estimation and monitoring. In: Knowledge Reference for National Forest Assessments. Rome: Food and Agriculture Organization of the United Nations; 2015. pp. 111–136.

31. Vieilledent G, Fischer FJ, Chave J, Guibal D, Langbour P, Gérard J. New formula and conversion factor to compute basic wood density of tree species using a global wood technology database. Am J Bot. 2018 Oct 1;105(10):1653–61.

32. Chave J, Andalo C, Brown S, Cairns MA, Chambers JQ, Eamus D, Fölster H, Fromard F, Higuchi N, Kira T, Lescure JP. Tree allometry and improved estimation of carbon stocks and balance in tropical forests. Oecologia. 2005 Aug;145(1):87-99.

33. Adams JD, Holmquist-Johnson C, Hanson L, Struckhoff MA. Orthoimagery and elevation data derived from UAS imagery for Palmyra Atoll, USA 2016 (ver.1.1, November 2020) [cited 2021 Jan 15]. US Geological Survey. Dataset available from https://www.sciencebase.gov/catalog/item/5d3b5502e4b01d82ce8d7898

34. Aalde H, Gonzalez P, Gytarsky M, Krug T, Kurz WA, Ogle S, et al. Forest land. In: 2006 IPCC Guidelines for National Greenhouse Gas Inventories; 2006. pp. 4.1-4.83.

35. Khalsa M. Operational plan Palmyra Atoll rainforest and reef resilience project PARP phase II: rainforest realignment. Santa Cruz (CA): Island Conservation; 2020. Final report.

36. Carlson CA, Hansell DA, Nelson NB, Siegel DA, Smethie WM, Khatiwala S, et al. Dissolved organic carbon export and subsequent remineralization in the mesopelagic and bathypelagic realms of the North Atlantic basin. Deep Res II [Internet]. 2010;57(16):1433–45. Available from: http://dx.doi.org/10.1016/j.dsr2.2010.02.013

37. FAO and ITPS. Global Soil Organic Carbon Map (GSOCmap); 2018 [Cited 2020 Jun 15]. Dataset available from: http://www.fao.org/soils-portal/data-hub/soil-maps-and-databases/global-soil-organic-carbon-map-gsocmap/en/

38. Spalding MD, Fox HE, Allen GR, Davidson N, Ferdaña ZA, Finlayson M, et al. Marine ecoregions of the world: A bioregionalization of coastal and shelf areas. Bioscience [Internet]. 2007 Jul 1;57(7):573–83. Available from: https://academic.oup.com/bioscience/article/57/7/573/238419

39. Morrison RJ. Pacific atoll soils: chemistry, mineralogy and classification. Atoll Research Bulletin. 1990.

40. Young HS, Raab TK, McCauley DJ, Briggs AA, Dirzo R. The coconut palm, Cocos nucifera, impacts forest composition and soil characteristics at Palmyra Atoll, Central Pacific. J Veg Sci. 2010 Dec;21(6):1058–68.

41. Chiti T, Grieco E, Perugini L, Rey A, Valentini R. Effect of the replacement of tropical forests with tree plantations on soil organic carbon levels in the Jomoro district, Ghana. Plant Soil. 2014 Feb;375(1–2):47–59.

42. Schroth G, D’Angelo SA, Teixeira WG, Haag D, Lieberei R. Conversion of secondary forest into agroforestry and monoculture plantations in Amazonia: consequences for biomass, litter and soil carbon stocks after 7 years. Forest ecology and management. 2002 Jun 28;163(1-3):131-50.

43. Silver WL, Ostertag R, Lugo AE. The potential for carbon sequestration through reforestation of abandoned tropical agricultural and pasture lands. Restor Ecol. 2000;8(4):394–407.

44. Brown S, Lugo AE. Effects of forest clearing and succession on the carbon and nitrogen content of soils in Puerto Rico and US Virgin Islands. Plant Soil. 1990;124(1):53–64.

45. Gibbs HK, Brown S, Niles JO, Foley JA. Monitoring and estimating tropical forest carbon stocks: Making REDD a reality. Environ Res Lett. 2007 Oct 1;2(4): 045023.

46. Hughes RF, Asner GP, Mascaro J, Uowolo A, Baldwin J. Carbon storage landscapes of lowland Hawaii: The role of native and invasive species through space and time. Ecol Appl. 2014;24(4):716–31.

47. Brown S, Lugo AE. The storage and production of organic matter in tropical forests and their role in the global carbon cycle. Biotropica. 1982 Sep 1:161-87.

48. Scharlemann JP, Tanner EV, Hiederer R, Kapos V. Global soil carbon: understanding and managing the largest terrestrial carbon pool. Carbon Management. 2014 Feb 1;5(1):81-91.

49. Montagnini F, Nair PKR. Carbon sequestration: an underexploited environmental benefit of agroforestry systems. In: Nair PKR, Rao MR, Buck LE, editors. New vistas in agroforestry. Advances in agroforestry, vol 1. Springer, Dordrecht; 2004. pp. 281 - 295.

50. Ranasinghe CS, Thimothias KSH. Estimation of carbon sequestration potential in coconut plantations under different agro-ecological regions and land suitability classes. J Natl Sci Found

Sri Lanka. 2012;40(1):77–93.

51. Carpenter SR, Mooney HA, Agard J, Capistrano D, DeFries RS, Díaz S, Dietz T, Duraiappah AK, Oteng-Yeboah A, Pereira HM, Perrings C. Science for managing ecosystem services: Beyond the Millennium Ecosystem Assessment. Proceedings of the National Academy of Sciences. 2009 Feb 3;106(5):1305-12.

52. Dickie IA, Yeates GW, St. John MG, Stevenson BA, Scott JT, Rillig MC, et al. Ecosystem service and biodiversity trade-offs in two woody successions. J Appl Ecol. 2011 Aug;48(4):926–34.

53. Eslamdoust J, Sohrabi H. Carbon storage in biomass, litter, and soil of different native and introduced fast-growing tree plantations in the South Caspian Sea. J For Res. 2018 Mar 1;29(2):449–57.

54. de Goeij J, van den Berg H, van Oostveen M, Epping E, van Duyl F. Major bulk dissolved organic carbon (DOC) removal by encrusting coral reef cavity sponges. Mar Ecol Prog Ser. 2008 Apr 7;357: 139 - 51.

55. Lorrain A, Houlbrèque F, Benzoni F, Barjon L, Tremblay-Boyer L, Menkes C, et al. Seabirds supply nitrogen to reef-building corals on remote Pacific islets. Sci Rep. 2017 Dec 16;7(1).

56. Goldberg WM. Atolls of the world: Revisiting the original checklist. Atoll Res Bull. 2016; 610.

57. Harries HC. The evolution, dissemination and classification of Cocos nucifera L. Bot Rev. 1978;44(3):265–319.

58. Gunn BF, Baudouin L, Olsen KM. Independent origins of cultivated coconut (Cocos nucifera L.) in the old world tropics. PLOS One. 2011 Jun 22;6(6): e21143.

59. Jeffery LR. Ecological restoration in a cultural landscape: conservationist and Chagossian approaches to controlling the 'coconut chaos' on the Chagos Archipelago. Human Ecology. 2014 Dec 1;42(6):999-1006.

60. Masselink G, Beetham E, Kench P. Coral reef islands can accrete vertically in response to sea level rise. Sci Adv [Internet]. 2020 Jun 1 [cited 2021 Feb 16];6(24):3656–66. Available from: http://advances.sciencemag.org/

61. Graham NA, Wilson SK, Carr P, Hoey AS, Jennings S, MacNeil MA. Seabirds enhance coral reef productivity and functioning in the absence of invasive rats. Nature. 2018 Jul;559(7713):250-3.

62. Benkwitt CE, Wilson SK, Graham NA. Seabird nutrient subsidies alter patterns of algal abundance and fish biomass on coral reefs following a bleaching event. Global change biology. 2019 Aug;25(8):2619-32.

63. Bossio DA, Cook-Patton SC, Ellis PW, Fargione J, Sanderman J, Smith P, Wood S, Zomer RJ, Von Unger M, Emmer IM, Griscom BW. The role of soil carbon in natural climate solutions. Nature Sustainability. 2020 May;3(5):391-8.

Supplementary Data

S1 Table. List of soil samples and data used in random forest model. Community type was assigned based on the value of the overlapping raster cell. Soil carbon percentages for each sample were binned as high (>20%), medium (10 – 20%), and low (<10%). Dry bulk density was assigned using the mean values collected in the field.

S2 Table. Basic wood density samples. Samples with the same sample ID were sampled from the same individual tree, with each sample (distinguished by sample number) designated individual discs measured as replicates.

S3 Table. Summary of quadratic mean of tree diameters for each species used in analysis with the number of trees sampled.

S4 Table. Table depicting results of health surveys for *H. foertherianum* seedlings, used to determine survival rate

S5 Table. Tables of pre and post transformation carbon values by islet. Islet areas are included.

S6 Table. Tables of pre and post transformation carbon values by major tree species. Note that the total values will not match those in S5 table as values were only calculated for the major tree species reported in Figure 2.

Figure 1 Click here to access/download;Figure;Figure1. Sampling sites.tif

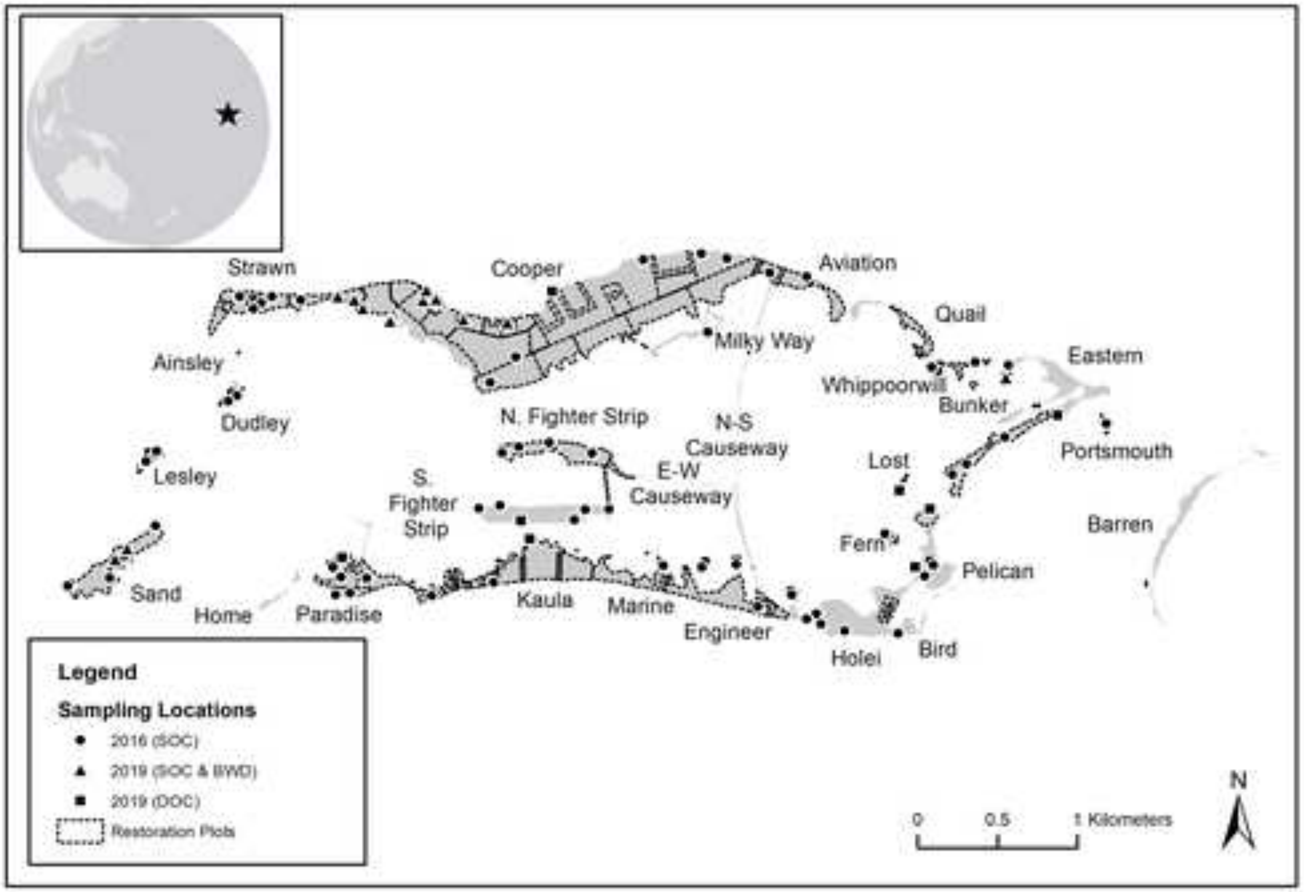

Figure 2

Click here to access/download;Figure;Figure 2.tif

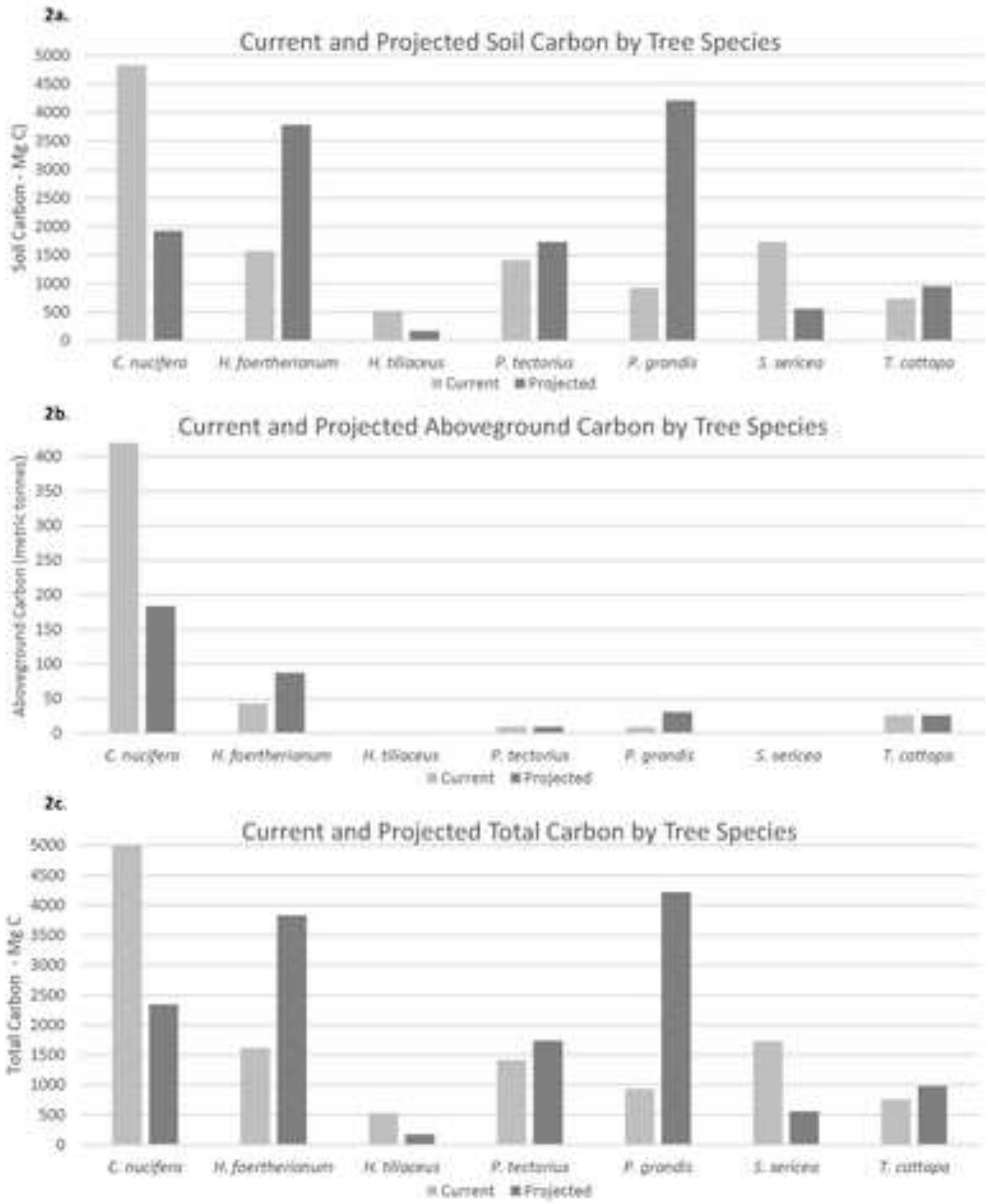

Figure 3

Click here to access/download;Figure;Figure 3.tif

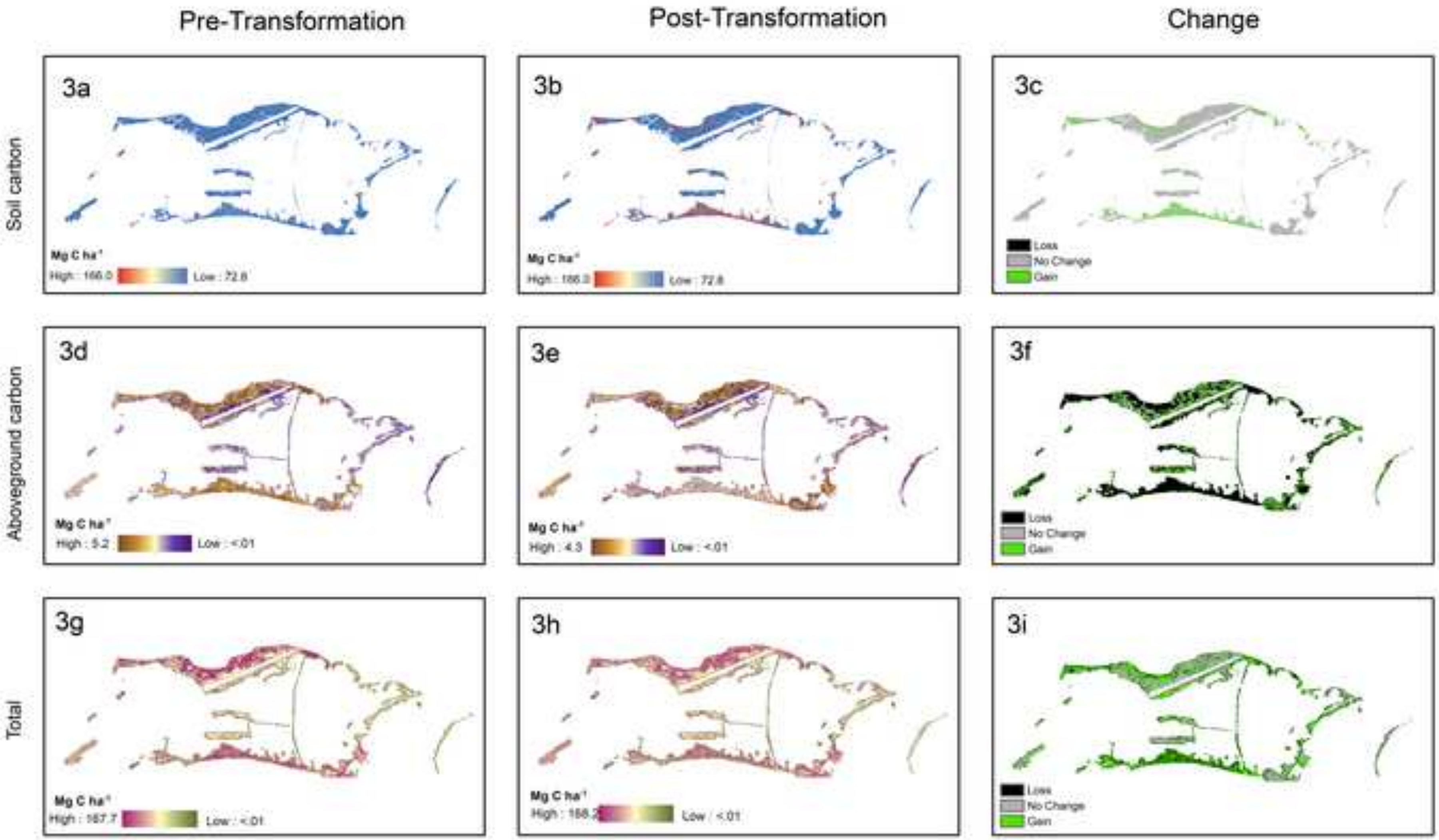

Figure 4

Click here to access/download;Figure;Figure 4. DOC sampling.tiff.tif

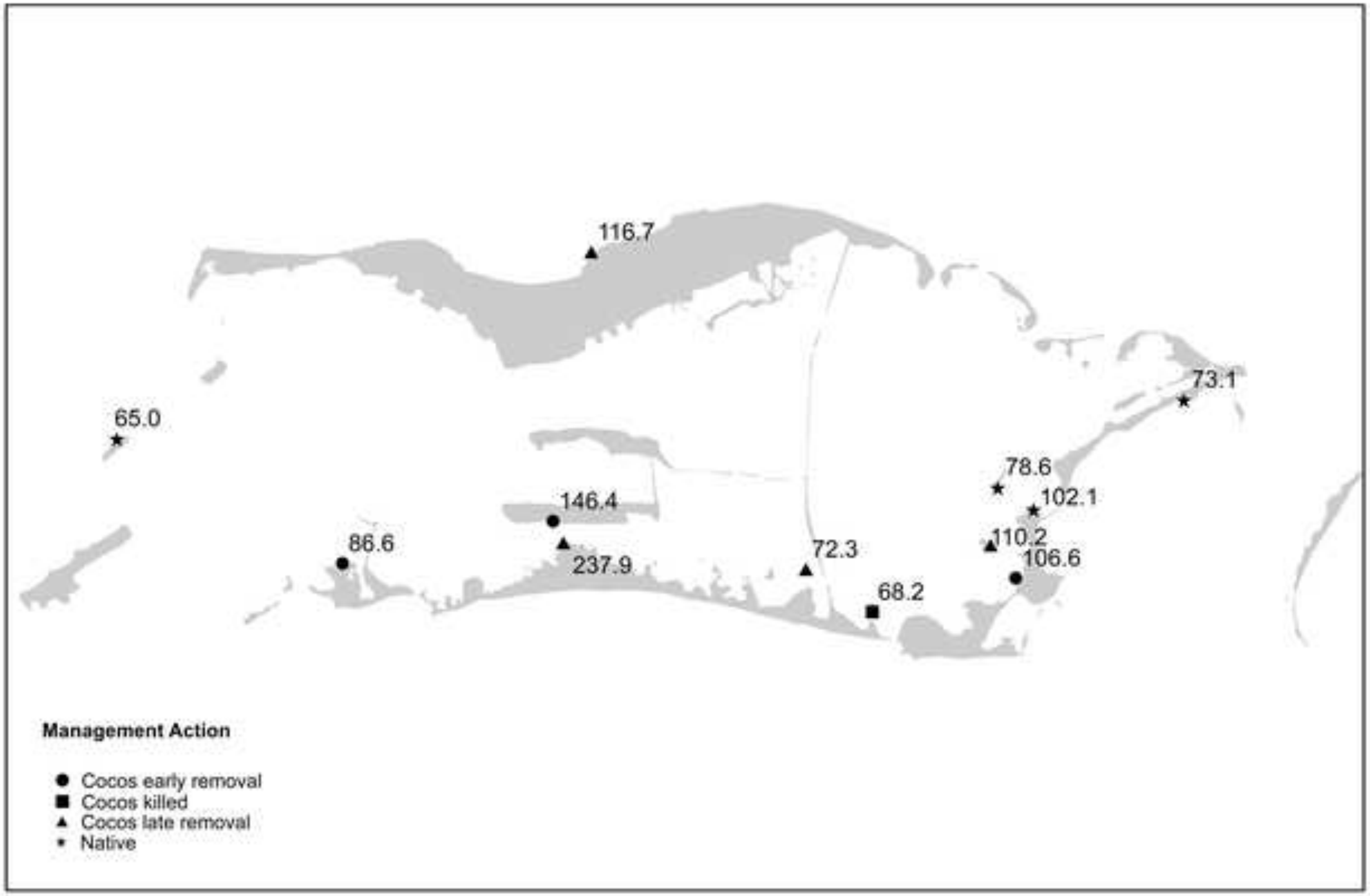

Figure 5

Click here to access/download;Figure;Figure 5.tif

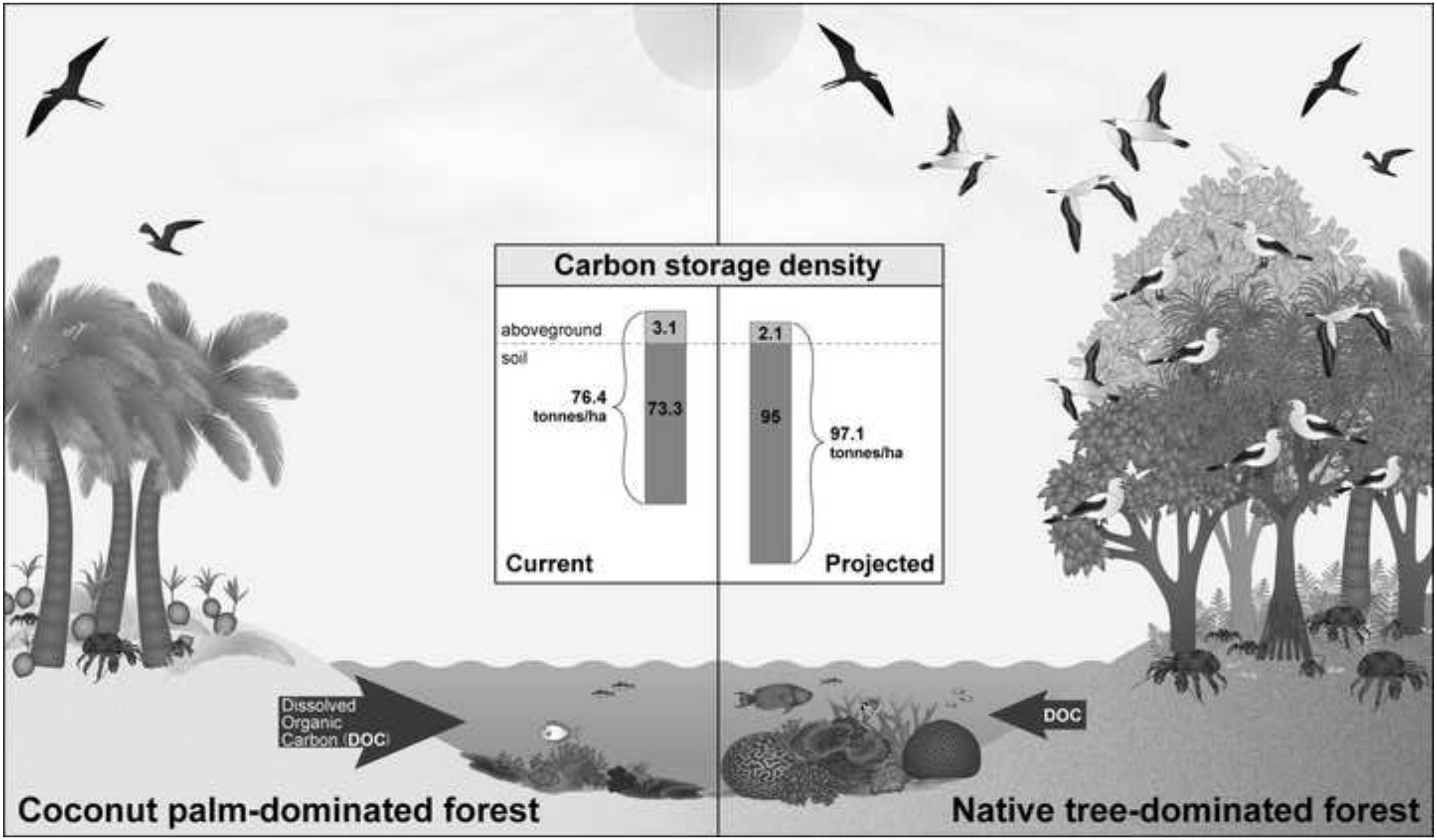

S1 Table

Click here to access/download
Supporting Information
S1 Table.docx

S2 Table

Click here to access/download
Supporting Information
S2 Table.docx

S3 Table

Click here to access/download
Supporting Information
S3 Table.docx

S4 Table

Click here to access/download
Supporting Information
S4 Table.docx

S5 Table

Click here to access/download
Supporting Information
S5 Table.docx

S6 Table

Click here to access/download
Supporting Information
S6 Table.docx
